# Association of apolipoproteins and lipoprotein(a) with metabolic syndrome: a systematic review and meta-analysis

**DOI:** 10.1186/s12944-023-01860-w

**Published:** 2023-07-07

**Authors:** Juan R. Ulloque-Badaracco, Ali Al-kassab-Córdova, Enrique A. Hernandez-Bustamante, Esteban A. Alarcon-Braga, Miguel Huayta-Cortez, Ximena L. Carballo-Tello, Rosa A. Seminario-Amez, Percy Herrera-Añazco, Vicente A. Benites-Zapata

**Affiliations:** 1 Facultad de Ciencias de La Salud, Universidad Peruana de Ciencias Aplicadas, Lima, Peru; 2 Centro de Excelencia en Investigaciones Económicas y Sociales en Salud, Universidad San Ignacio de Loyola, Lima, Peru; 3 Grupo Peruano de Investigación Epidemiológica, Unidad Para La Generación Y Síntesis de Evidencias en Salud, Universidad San Ignacio de Loyola, Lima, Peru; 4 Sociedad Científica de Estudiantes de Medicina de La Universidad Nacional de Trujillo, Trujillo, Peru; 5 Universidad Privada del Norte, Trujillo, Peru; 6 Red Peruana de Salud Colectiva, Lima, Peru; 7 Vicerrectorado de Investigación, Unidad de Investigación Para La Generación Y Síntesis de Evidencias en Salud, Universidad San Ignacio de Loyola, Lima, Peru

**Keywords:** Metabolic syndrome, Apolipoproteins, Lipoprotein(a)

## Abstract

**Background & aims:**

Apolipoproteins and lipoprotein(a) are associated with various cardiometabolic diseases, including insulin resistance, diabetes mellitus, hypertension, dyslipidemia, among others. This systematic review and meta-analysis was conducted to evaluate the association of these markers with metabolic syndrome (MetS).

**Methods:**

We ran a systematic search through PubMed, Scopus, Embase, Ovid/Medline, and Web of Science on March 15, 2023. No language or date restrictions were applied. The only synthesised effect measure reported was the odds ratio (OR) with its corresponding 95% confidence interval (95% CI). We utilised the random-effects model for the quantitative synthesis.

**Results:**

We analysed 50 studies (*n* = 150 519) with different definitions for MetS. Increased ApoB values were associated with MetS (OR = 2.8; 95% CI: 2.44–3.22; *p* < 0.01, I^2^ = 99%). Decreased ApoA1 values were associated with MetS (OR = 0.42; 95% CI: 0.38–0.47; *p* < 0.01, I^2^ = 99%). Increased values of the ApoB/ApoA1 ratio were associated with MetS (OR = 4.97; 95% CI: 3.83–6.44; *p* < 0.01, I^2^ = 97%). Decreased values of Lp(a) were associated with MetS (OR = 0.89; 95% CI: 0.82–0.96; *p* < 0.01; I^2^ = 92%).

**Conclusions:**

Increased values of ApoB and ApoB/ApoA1 ratio are associated with MetS, while decreased values of ApoA1 and Lp(a) are associated with MetS. These findings suggest that these lipid markers may serve as potential indicators for identifying subjects at risk of developing MetS. However, further research is required to elucidate the underlying mechanisms of these associations.

**Supplementary Information:**

The online version contains supplementary material available at 10.1186/s12944-023-01860-w.

## Introduction

Metabolic syndrome (MetS) is associated with a fivefold increase in the risk of diabetes mellitus, a twofold higher risk of cardiovascular events, and a 1.5-fold higher risk of all-cause mortality [1, 2]. Consequently, the increase in its prevalence is a public health concern. Indeed, although estimates of the prevalence of MetS vary according to the criteria used for its definition, certain studies indicate an increase in its prevalence in some countries. In the United States, the prevalence of MetS increased from 28.23% to 37.09% between 1999 and 2018 [3], and in Mexico increased from 40.2% to 56.31% between 2006 and 2018, respectively [4].

Apolipoproteins are constituents of high-density lipoproteins (HDL) and triglyceride-rich lipoproteins [5]. Due to their potential effects and prominence in different pathologies, apolipoproteins have been extensively investigated as predictors of clinical outcomes [6, 7]. For example, in the case of Apolipoprotein A1 (ApoA1), some studies found that alteration in its levels was associated with cardiovascular outcomes and it has also been evaluated as a diagnostic and prognostic marker for some cancers [8, 9]. In addition to these, other studies have associated apolipoprotein values with the development of some metabolic diseases [10].

Circulating apolipoprotein levels reflect the number of lipoprotein particles, rather than the concentration of cholesterol [11]. In this regard, the level of Apolipoprotein B (Apo B) reflects the number of triglyceride-rich Very Low Density Lipoprotein (VLDL) particles and the number of Low Density Lipoprotein (LDL) particles [11]. Therefore, it places more emphasis on the number of small and dense LDL particles than the usual measurement of LDL cholesterol [11]. Similarly, the level of ApoA1 corresponds to the quantity of HDL particles; therefore, apolipoproteins taken individually or the ratio of ApoB and ApoA1 (ApoB/A1 ratio) would theoretically serve as optimal markers of lipid abnormalities associated with insulin resistance and MetS [11]. Several studies have found an independent association between ApoA1, ApoB and ApoB/A1 ratio values with this syndrome [12–15]. However, to the best of our knowledge, there has been no systematic review of the available evidence regarding these associations. Therefore, the objective of this study was to conduct a systematic review and meta-analysis to synthesise the evidence on the association between ApoA1, ApoB, ApoB/A1 ratio and lipoprotein(a) [Lp(a)] values with MetS.

## Methods

### Registration and reporting

In the development of this systematic review, we adhered to guidelines outlined in the Preferred Reporting Items for Systematic Reviews and Meta-Analyses statement [16]. A version of this systematic review’s protocol has been uploaded to the International Prospective Register of Systematic Reviews (PROSPERO) [CRD42023416427].

### Search strategy and databases

The search strategy was designed using the Peer Review of Electronic Search Strategies Checklist [17]. There were no limitations regarding language or date. We performed a systematic search of various databases including PubMed, Scopus, Embase, Ovid/Medline, and Web of Science on March 15, 2023. We also reviewed the reference list of the selected studies and manually searched preprint databases. Additionally, the reference lists of the included studies were thoroughly examined and we conducted a manual search of preprint databases. For details of the complete search strategy, please refer to Supplementary Material (Table S1).

### Study selection and data extraction

We selected studies with the following characteristics: cohort/case–control/cross-sectional studies that evaluated the association between ApoB, ApoA1, ApoB/ApoA1 ratio and Lp (a), and MetS in adult patients (> 18 years). We excluded: duplicated studies, scoping reviews, systematic reviews, narrative reviews and conference abstracts.

The studies retrieved from the systematic search were uploaded to the data management software Rayyan QCRI. After removing duplicated studies, four authors (J.R.U-B, M.A.H-C, X.L.C-T and R.A.S-A) independently assessed the title/abstract of each study according to the selection criteria. Once the relevant literature was identified, two reviewers (M.A.H-C and X.L.C-T) independently assessed the full text of each article. The studies that did not comply with the entire selection criteria were excluded from the review. In case of missing information, we contacted the authors. Any discrepancies were resolved through discussion and consensus between the two reviewers.

For extracting data, we employed a standardised data collection sheet created in Google Sheets©. The data were gathered independently by two authors (E.A.H-B and E.A.A-B) from each study and included the following details: title, study location, first author, publication date, study design sample size, age, sex, definition of MetS, ApoB levels(mg/dL), ApoA1 levels(mg/dL), ApoB/ApoA1 ratio levels, Lp(a) levels(mg/dL) and assay technique. For articles published in a language other than English, we proceeded to translate them using online translation tools.

### Risk of bias and publication bias

Two authors (J.R.U-B and E.A.A-B) independently performed the risk of bias assessment. The Newcastle–Ottawa Scale (NOS) was used for cohort and case-controls studies, whereas an adjusted version of the NOS for cross-sectional studies (NOS-CS) was applied to this type of study. A score of ≥ 7 stars was considered indicative of a low risk of bias, while a score of < 7 stars was considered indicative of a high risk of bias. To assess publication bias, we employed funnel plots and the Begg test.

### Data synthesis

The statistical analysis was performed in STATA 17.0 © and Review Manager v.5.4 (The Cochrane Collaboration, Copenhagen, Denmark). For all meta-analyses, we employed a random-effects model (Restricted Maximum Likelihood). All the effect measures were reported as the odds ratios (OR) with their corresponding 95% confidence intervals (CI). Any other effect measure was transformed into OR. Using Hozo's method, we converted median values and their interquartile ranges were converted into means and their corresponding standard deviations (SD) [18]. We also transformed standard mean differences into the natural logarithm of the OR (lnOR) and its standard error using Chinn's method [19]. We used the Cochran's Q test and the I^2^ statistic to assess between-study heterogeneity, in which I^2^ ≥ 60% for the I^2^ test and a *p*-value < 0.05 for the Cochran's Q test indicated high heterogeneity. Subgroup analyses were conducted according to continent, sex, assay method, and MetS diagnostic criteria. In the sensitivity analysis, we excluded studies with a high risk of bias.

## Results

### Eligible studies

**Fig. 1:**
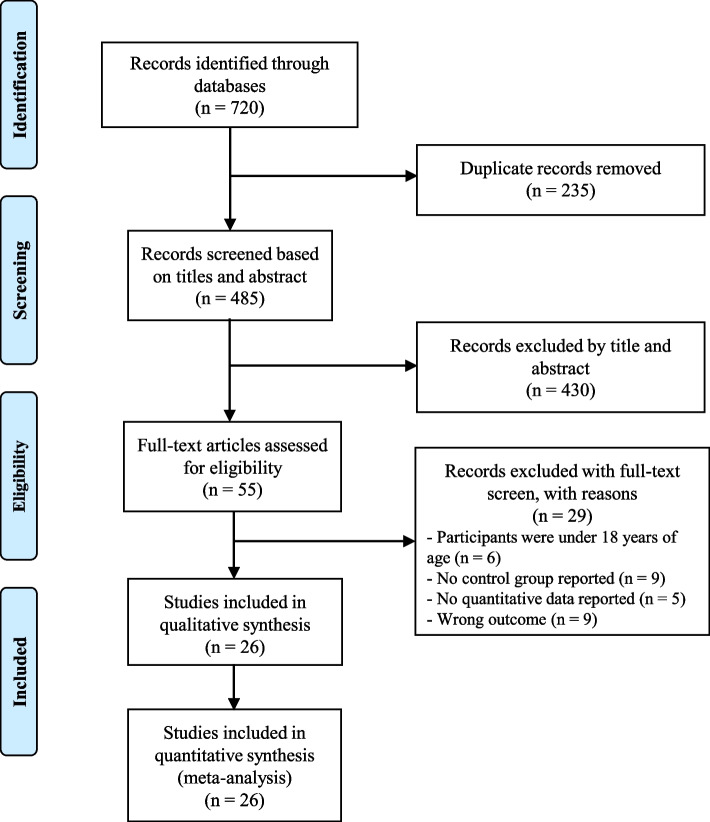
PRISMA flow diagram of study selection process

In the search strategy, a total of 2262 studies were identified. After removing duplicates and reviewing titles and abstracts, 153 studies met the selection criteria and advanced to the full-text reading phase. Upon reading the full text of each article, 50 studies were deemed eligible for inclusion in the qualitative synthesis and meta-analysis [11, 13, 20–67]. Figure 1 represents the flow chart of the selection process.

### Study characteristics and risk of bias assessment

A total of 150 519 participants were included, of which 62 083 were female and 77 958 were male. Six studies (*n* = 10,486) did not report the sex of the included participants. Ultimately, fifty studies were included, of which 40 had a cross-sectional design, 7 were case–control studies, and 3 were cohort studies. Six studies [21, 33, 49, 50, 52, 66] analysed the association between two different participant groups, resulting in a total of 56 included studies. Characteristics from all included studies are summarised in Table 1.

**Table 1:** Characteristics of the included studies

Author	Year	Country	Median/mean/Range age (IQR/SD)	Participants (male/female)	Comorbidities (n)				MetS definition criteria	Marker analyzed	Marker mean (SD) in patients with MetS	Marker mean (SD) in patients without MetS	Assay Method	Odds Ratio (95% CI)
					Obesity	CHD	HT	DM						
**Bonora E et al**	2003	Italy	MetS: 60.7(11.6) Control: 58.5(11.4)	888(446/442)	NR	NR	551	201	NCEP-ATP III	ApoA1	150 (30)	170(30)	IN	NR
										ApoB	150 (40)	120(30)	IN	NR
**Muntner P et al**	2004	United States of America	≥ 20	7347(NR/NR)	NR	NR	NR	NR	NCEP-ATP III	ApoA1	NR	NR	IN	2.27 (1.30–3.96)
										ApoB	NR	NR	IN	2.97 (2.03–4.34)
										Lp(a)	NR	NR	IN	0.78 (0.54–1.12)
**Panagiotakos D et al**	2004	Greece	MetS: 55(13) Control: 45(13)	2282(1126/1154)	466	NR	897	NR	NCEP-ATP III	ApoA1	146.45 (71.77)	158.04(26.1)	IN	NR
										ApoB	123.95 (28.42)	105.35(41.27)	IN	NR
										Lp(a)	17.65 (22.07)	19.84(28.95)	IN	NR
**Blatter M et al**	2005	Switzerland	MetS: 62.1(8.1) Control: 59.6(9.6)	773(558/215)	NR	606	NR	145	WHO 1999	ApoA1	91 (17)	97 (22)	IN	NR
										ApoB	98 (21)	96 (23)	IN	NR
**Guven A et al**	2005	Turkey	MetS:38(25–48) Control:35(26–48)	101(49/52)	NR	NR	NR	NR	NCEP-ATP III	Lp(a)	51.1 (85.48)	25.25 (42.07)	IN	NR
**Lind L et al**	2005	Sweden	50–70	1826(1826/0)	NR	NR	NR	NR	NCEP-ATP III	ApoA1	126(19)	146(25)	RIA	NR
										ApoB	137(28)	122(27)	RIA	NR
										ApoB/ApoA1 ratio	1.11(0.27)	0.86(0.24)	-	NR
**Cankurtaran M et al**	2006	Turkey	71.8(6.3)	1255(466/789)	574	427	1117	732	NCEP-ATP III	ApoA1	NR	NR	IN	0.997 (0.992–1.002)
										ApoB	NR	NR	IN	1.005 (1–1.01)
										Lp(a)	NR	NR	IN	1 (0.992–1.007)
**Sierra-Johnson J et al**	2006	United States of America	46.8(19)	2954 (1516/1448)	NR	NR	NR	NR	NCEP-ATP III	ApoB/ApoA1 ratio	0.91 (0.2)	0.69 (0.2)	–	NR
**Al-Daghri N et al**	2007	Saudi Arabia	MetS: 48.82(12.22) Control: 41.05(10.15)	581(294/287)	NR	154	NR	186	IDF	ApoA1	106 (144)	83 (26)	IT	NR
**Pei W et al**	2007	China	≥ 20	560(268/292)	NR	NR	NR	NR	NCEP-ATP III	ApoA1	NR	NR	IT	0.951 (0.937–0.965)
										ApoB	NR	NR	IT	1.064 (1.048–1.080)
**De Souza J et al**	2008	France	MetS: 53(10) Control: 46(12)	23(23/0)	NR	NR	NR	NR	NCEP-ATP III	ApoA1	136(23)	159(16)	IN	NR
										ApoB	133(24)	85(19)	IN	NR
**Hye J et al. (A)**	2008	South Korea	20–78	1671(1671/0)	NR	NR	NR	NR	NCEP-ATP III	ApoB	99.7 (22.6)	86.5(23.5)	IT	NR
**Hye J et al. (B)**	2008	South Korea	20–78	1664(0/1664)	NR	NR	NR	NR	NCEP-ATP III	ApoB	100.1 (23.5)	77.4(24.9)	IT	NR
**Kotani K et al**	2008	Japan	MetS: 71(6.6) Control: 73.2(7.6)	182(62/120)	NR	NR	NR	NR	NCEP-ATP III	Lp(a)	NR	NR	IT	1.01 (0.99–1.03)
**Onat A et al**	2008	Turkey	56.8(11.3)	1309(608/701)	NR	NR	NR	NR	NCEP-ATP III	Lp(a)	NR	NR	IN	0.62(0.47–0.81)
**Pitsavos C et al**	2008	Greece	MetS: 51(13) Control: 43(13)	3042(1518/1524)	438	NR	622	NR	NCEP-ATP III	ApoA1	NR	NR	IN	0.94 (0.90–0.98)
										ApoB	NR	NR	IN	1.09(1–1.18)
										ApoB/ApoA1 ratio	NR	NR	–	2.3(1.65–3.2)
**Dullaart R et al**	2009	Netherlands	MetS: 59(10) Control: 55(9)	79(43/36)	NR	NR	NR	NR	NCEP-ATP III	ApoA1	130 (24)	147 (20)	IT	NR
										ApoB	99 (22)	93 (25)	IT	NR
**Boumaiza I et al**	2010	Tunisia	MetS: 62.6(9.3) Control: 59.7(10.2)	192(NR/NR)	NR	113	91	72	IDF	ApoA1	125 (4.2)	154 (39)	IN	NR
										ApoB	NR	NR	IN	2.80 (1.50–5.21)
										ApoB/ApoA1 ratio	1.58 (0.61)	0.97 (0.41)	–	NR
**Mattsson N et al**	2010	Finland	24–39	2183(NR/NR)	NR	NR	NR	NR	IDF	ApoA1	138 (22)	150 (25)	IT	NR
										ApoB	131 (26)	101 (23)	IT	NR
										ApoB/ApoA1 ratio	0.97 (0.21)	0.69(0.2)	–	NR
**Park J et al**	2010	South Korea	MetS: 54.64(10.84) Control: 53.99(11.28)	658(327/331)	NR	NR	NR	NR	NCEP-ATP III	ApoB/ApoA1 ratio	0.75 (0.25)	0.69 (0.41)	–	NR
**Riediger N et al**	2010	Canada	≥ 18	475(NR/NR)	NR	NR	201	140	NCEP-ATP III	ApoA1	113 (17)	120 (18)	IN	NR
										ApoB	102 (26)	80 (23)	IN	NR
										ApoB/ApoA1 ratio	0.9 (0.22)	0.65 (0.2)	–	NR
**Belfki H et al**	2011	Tunisia	MetS: 54.9(11) Control: 50.4(13.2)	330(94/236)	NR	NR	NR	NR	NCEP-ATP III	ApoA1	149 (28)	167(31)	IT	NR
										ApoB	97 (23)	80(19)	IT	NR
										ApoB/ApoA1 ratio	0.67 (0.19)	0.49(0.13)	–	NR
**Hee C et al. (A)**	2012	South Korea	51.8(10.9)	7867(7867/0)	NR	297	2438	902	NCEP-ATP III	ApoB/ApoA1 ratio	NR	NR	–	2.43(2.17–2.72)
**Hee C et al. (B)**	2012	South Korea	51.9(9)	3073(0/3073)	NR	108	724	179	NCEP-ATP III	ApoB/ApoA1 ratio	NR	NR	–	3.84(3.13–4.71)
**Won D et al**	2012	South Korea	52.9(8.2)	244(159/85)	NR	NR	100	76	NCEP-ATP III	ApoA1	128 (20.3)	137.4(18.5)	IT	NR
										ApoB	101.7 (24.2)	88.9(20.4)	IT	NR
**Li Y et al**	2013	China	MetS: 27.36(4.79) Control: 26.68(4.18)	185(0/185)	NR	NR	NR	NR	IDF	ApoA1	101 (20)	125(30)	IT	NR
										ApoB	93 (16)	72(17)	IT	NR
										ApoB/ApoA1 ratio	0.89 (0.21)	0.6(0.18)	–	NR
**Sung K et al**	2013	South Korea	42.42(6.91)	14,283(12,031/2252)	NR	102	1704	539	HDM	Lp(a)	NR	NR	IT	0.96(0.84–1.09)
**Won S et al. (A)**	2013	South Korea	MetS: 51.6(9.2) Control: 50.5(9.4)	23,010(23,010/0)	NR	NR	NR	NR	HDM	ApoA1	132.2(24.1)	139.2(23.4)	IT	NR
										ApoB	102.7(21.5)	93(20.9)	IT	NR
										ApoB/ApoA1 ratio	0.8(0.21)	0.69(0.2)	–	NR
**Won S et al. (B)**	2013	South Korea	MetS: 56.9(8.9) Control: 49.2(8.9)	18,811(0/18811)	NR	NR	NR	NR	HDM	ApoA1	137.9(23.3)	152.6(25)	IT	NR
										ApoB	103.8(22.3)	85.8(21.3)	IT	NR
										ApoB/ApoA1 ratio	0.77(0.21)	0.58(0.18)	–	NR
**Jing F et al**	2014	China	MetS: 55.7(12.68) Control: 49.27(15.14)	8120(3781/4339)	NR	179	2469	600	IDF	ApoB/ApoA1 ratio	NR	NR	–	4.3 (3.48–5.31)
**Makaridze Z et al. (A)**	2014	Georgia	18–80	869(869/0)	NR	NR	NR	NR	NCEP-ATP III	ApoB/ApoA1 ratio	NR	NR	–	1.18(0.58–2.4)
**Makaridze Z et al. (B)**	2014	Georgia	18–80	653(0/653)	NR	NR	NR	NR	NCEP-ATP III	ApoB/ApoA1 ratio	NR	NR	–	1.75(0.78—3.9)
**Prasad M et al**	2014	India	43(10)	1000(701/299)	NR	24	307	163	HDM	Lp(a)	NR	NR	IT	0.7(0.5–1)
**Savinova O et al**	2014	United States of America	44.96(12.3)	70(42/28)	NR	NR	14	NR	NCEP-ATP III	ApoA1	98.86(19.1)	117.76(14.9)	Electrophoresis	NR
										ApoB	87.96(23.2)	69.86(17.4)	Electrophoresis	NR
**Chou YL et al. (A)**	2015	China	39.8(15.61)	1531(1531/0)	192	NR	201	57	HDM	ApoB/ApoA1 ratio	NR	NR	–	2.86 (1.6–5.1)
**Chou YL et al. (B)**	2015	China	39.8(15.61)	1811(0/1811)	155	NR	126	65	HDM	ApoB/ApoA1 ratio	NR	NR	–	2.01 (1.67–2.41)
**Lim Y et al**	2015	South Korea	MetS: 58.6(12.8) Control: 58.1(13.4)	912(516/396)	NR	NR	NR	912	NCEP-ATP III	ApoB	104.5 (53.3)	87.7(33.7)	IN	NR
**Barkas F et al**	2016	Greece	MetS: 57(50–64) Control: 52(43–61)	738(314/424)	NR	NR	NR	NR	HDM	ApoA1	141 (30)	155(30)	IN	NR
										ApoB	122 (25)	123(31)	IN	NR
										Lp(a)	10.65 (9.92)	14.97(14.59)	IN	NR
**Gentile M et al**	2016	Italy	MetS: 64.1(7.4) Control: 62.5(8.7)	222(0/222)	NR	NR	NR	NR	AHA/NLBI	ApoB	110 (20)	100 (20)	IT	NR
										Lp(a)	19.1 (22.1)	27.9 (29.7)	ELISA	NR
**Borja M et al**	2017	United States of America	MetS: 47(10) Control: 45(12)	74(44/30)	NR	NR	13	NR	HDM	ApoA1	98 (19)	116 (14)	Electrophoresis	NR
**Sreckovic B et al**	2017	Serbia	30–75	76(NR/NR)	NR	NR	NR	NR	NCEP-ATP III	ApoB	108 (29)	91 (25)	IT	NR
**Vaverková H et al**	2017	Czech Republic	45.6(14)	607(295/312)	NR	NR	NR	NR	HDM	Lp(a)	NR	NR	IT	0.309 (0.184–0.516)
**Andrea G et al**	2018	India	MetS: 55.6(5.66) Control: 52.98(6.76)	100(53/47)	NR	NR	NR	NR	AHA/NLBI	ApoA1	89.06 (24.1)	173.13(24.11)	IN	NR
										ApoB/ApoA1 ratio	1.93 (1.18)	0.48(0.13)	–	NR
**Mokhsin A et al. (A)**	2018	Malaysia	MetS: 49.53(11.7) Control: 40(14.69)	1177(465/712)	NR	NR	1246	98	IDF	Lp(a)	6 (4)	6 (4)	IT	NR
**Mokhsin A et al. (B)**	2018	Malaysia	Mets: 30.56(11.1) Control: 31.29(11)	150(79/71)	NR	NR	86	3	IDF	Lp(a)	23 (26)	22 (22)	IT	NR
**Boiko A et al**	2019	Russia	35(25.5–42.5)	53(27/26)	NR	NR	NR	NR	IDF	ApoA1	48.69 (16.07)	56.3 (24.25)	IT	NR
**Du R et al**	2019	China	58.5(9.7)	10,340(3940/6400)	NR	NR	NR	NR	NCEP-ATP III	ApoB	NR	NR	CLIA	1.49 (1.43–1.55)
**Jun J et al**	2019	South Korea	51.6(NR)	10,150(6141/4009)	NR	NR	NR	NR	NCEP-ATP III	Lp(a)	NR	NR	IT	0.63(0.49–0.80)
**Reynoso-Villalpando G et al**	2019	Spain	MetS: 65(9.94) Control: 68(1.3)	100(65/35)	50	NR	80	100	NCEP-ATP III	ApoA1	132.45 (15.91)	145.96(17.5)	IT	NR
										ApoB	84.84 (20.2)	75.65(19.03)	IT	NR
										ApoB/ApoA1 ratio	0.61 (0.16)	0.5(0.16)	–	NR
										Lp(a)	28.99 (36)	33.88(40.11)	IT	NR
**Wu X et al**	2019	China	≥ 40	10,336(3944/6392)	NR	1270	6259	1488	NCEP-ATP III	Lp(a)	NR	NR	IT	1.67(1.52–1.83)
**Cardoso-Saldaña G et al**	2020	Mexico	MetS: 54.1(8.8) Control: 52.7(9.6)	953(481/472)	NR	NR	NR	NR	NCEP-ATP III	Lp(a)	4.7 (5.11)	6.85 (7.85)	IN	NR
**Nurtazina A et al**	2020	Kazakhstan	25–75	704(314/390)	NR	158	408	NR	IDF	ApoB/ApoA1 ratio	NR	NR	–	4.73 (3.01–7.43)
**Rohit A et al**	2020	India	21–80	150(90/60)	NR	NR	NR	NR	AHA/NLBI	ApoA1	113.91 (24.22)	146.94(12.82)	IN	NR
										ApoB	126.09 (37.02)	85.65(18.85)	IN	NR
										Lp(a)	19.33 (7.64)	19.58(4.75)	IN	NR
**He H et al**	2021	China	27.25(3)	957(0/957)	NR	NR	NR	NR	IDF	ApoB/ApoA1 ratio	NR	NR	–	8.7 (6.1–12.4)
**Sharan H et al**	2022	Nepal	MetS: 54.89(8.93) Control: 54.63(9.44)	213(NR/NR)	NR	NR	NR	NR	NCEP-ATP III	ApoA1	101.36 (11.75)	116.52(12.22)	IT	NR
										ApoB	115.22 (25.03)	81.77(16.13)	IT	NR
										ApoB/ApoA1 ratio	1.14 (0.27)	0.7 (0.15)	–	NR
**Wang W et al**	2022	China	53.4(7.5)	605(304/301)	NR	NR	215	605	CDS	ApoA1	96 (19)	114 (20)	IT	NR

*AHA/NHLBI* American Heart Association/National Heart, Lung, and Blood Institute, *ApoB* Apolipoprotein B, *ApoA1* Apolipoprotein A1, *CDS* Chinese Diabetes Society, *HDM* Harmonized Definition of MetS, *MetS* Metabolic Syndrome, *NCEP-ATP III* National Cholesterol Education ProgramAdult Treatment Panel III, *Lp(a)* Lipoprotein(a), *CLIA* Chemiluminescence immunoassay, *IT* Immunoturbidimetric, *IN* Immunonephelometry, *ELISA* Enzyme-linked immune-sorbent assay, *IDF* International Diabetes Federation, *NR* Not Reported, *RIA* Radioimmunoassay, *WHO* World Health Organization

The distribution of MetS definitions used in the studies was as follows: 32 studies used the National Cholesterol Education Programme-Adult Treatment Panel III (NCEP-ATP III) criteria [68], 10 studies used the International Diabetes Federation criteria [69], 9 studies met the Harmonised Definition of MetS (HDM) criteria [70], 3 studies met the American Heart Association/National Heart Lung and Blood Institute (AHA/NHLBI) criteria [71], 1 study fulfilled the Chinese Diabetes Society (CDS) criteria [72], and 1 study met the World Health Organization (WHO 1999) criteria [73]. The definitions of MetS according to each criterion are detailed in Supplementary Table S2. Regarding the risk of bias assessment, a total of 46 studies were classified as having a low risk of bias, while 10 studies were classified as having a high risk of bias (Supplementary Table S3).

### Association between ApoB levels and the presence of MetS

**Fig. 2:**
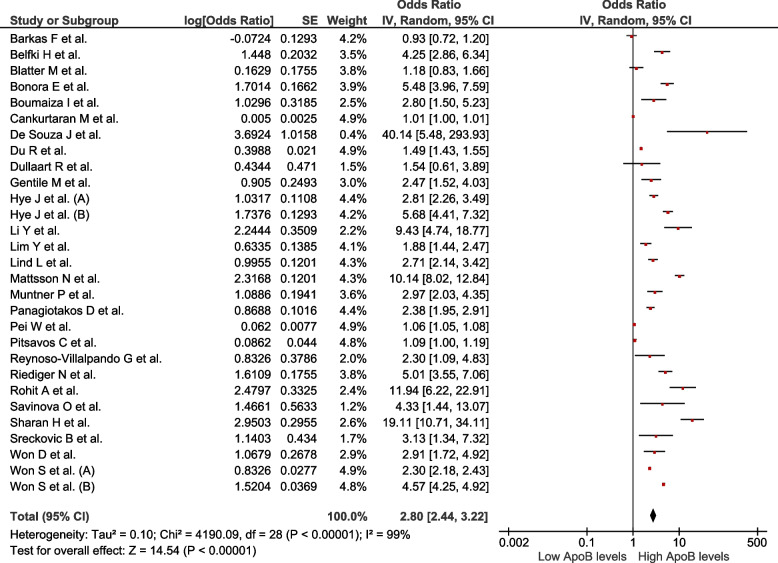
Association between ApoB and MetS

Twenty-nine studies assessed this association (*n* = 79,661). Increased ApoB values were associated with MetS (OR = 2.8; 95% CI: 2.44–3.22; *p* < 0.01, I^2^ = 99%) (Fig. 2). Subgroup analysis was performed according to assay method (Supplementary Figure S1), MetS diagnostic criteria (Supplementary Figure S2), sex (Supplementary Figure S3), continent (Supplementary Figure S4), and study design (Supplementary Figure S5). High heterogeneity was observed in all subgroups, indicating that the association persisted across various subgropus. In the sensitivity analysis (Supplementary Figure S6), after eliminating studies at high risk of bias, the association persisted with high heterogeneity (OR = 3.29; 95% CI: 2.63–4.13; *p* < 0.01, I^2^ = 98%).

### Association between ApoA1 levels and the presence of MetS

**Fig. 3:**
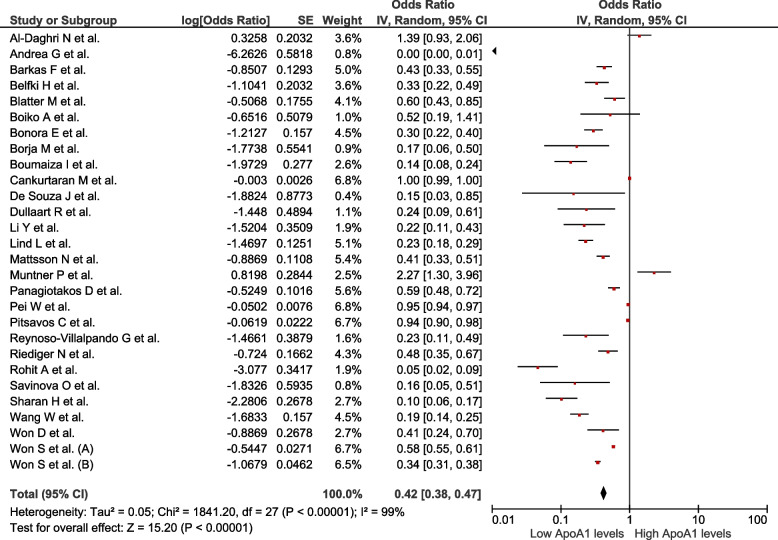
Association between ApoA1 and MetS

Twenty-eight studies assessed this association (*n* = 66 189). Reduced ApoA1 values were associated with MetS (OR = 0.42; 95% CI: 0.38–0.47; *p* < 0.01, I^2^ = 99%) (Fig. 3). Subgroup analysis was performed according to assay method (Supplementary Figure S7), MetS diagnostic criteria (Supplementary Figure S8), sex (Supplementary Figure S9), continent (Supplementary Figure S10), and study design (Supplementary Figure S11). In all subgroups, except for the subgroup of American studies, the association remained significant despite the presence of high heterogeneity. In the sensitivity analysis (Supplementary Figure S12), after eliminating studies at high risk of bias, the association persisted with high heterogeneity (OR = 0.26; 95% CI: 0.18–0.38; *p* < 0.01, I^2^ = 97%).

### Association between ApoB/ApoA1 ratio levels and the presence of MetS

**Fig. 4:**
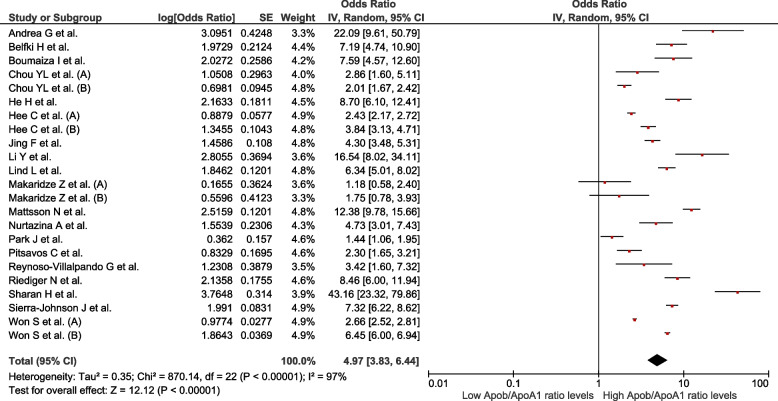
Association between ApoB/ApoA1 ratio and MetS

Twenty-three studies assessed this association (*n* = 79 664). Increased values of the ApoB/ApoA1 ratio were associated with MetS (OR = 4.97; 95% CI: 3.83–6.44; *p* < 0.01, I^2^ = 97%) (Fig. 4). The subgroup analysis was performed according to MetS diagnostic criteria (Supplementary Figure S13), sex (Supplementary Figure S14), continent (Supplementary Figure S15), and study design (Supplementary Figure S16). In all subgroups, the association remained with high heterogeneity. In the sensitivity analysis (Supplementary Figure S17), the association continued to be observed despite the exclusion of studies considered to have a high risk of bias. However, high heterogeneity remained a characteristic of the association (OR = 5.42; 95% CI: 3.88–7.56; *p* < 0.01, I^2^ = 96%).

### Association between Lp(a) levels and the presence of MetS

**Fig. 5:**
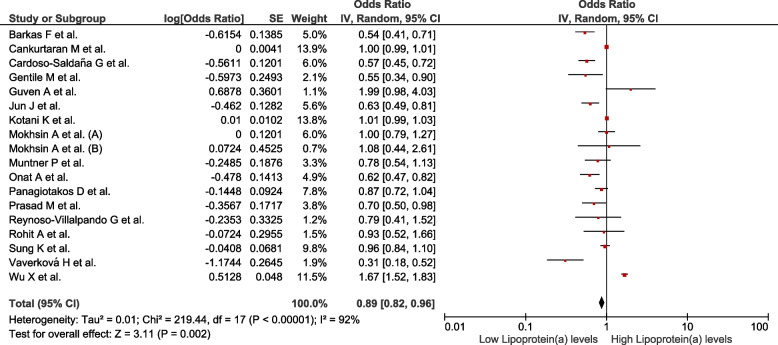
Association between lipoprotein(a) and MetS

Eighteen studies assessed this association (*n* = 52 342). Decreased Lp (a) values were associated with MetS (OR = 0.89; 95% CI: 0.82–0.96; *p* < 0.01; I^2^ = 92%) (Fig. 5). The subgroup analysis was performed according to assay method (Supplementary Figure S18), MetS diagnostic criteria (Supplementary Figure S19), continent (Supplementary Figure S20), and study design (Supplementary Figure S21). The studies conducted in Europe (OR = 0.71; 95% CI: 0.55–0.93; *p* = 0.01; I^2^ = 89%), those using immunonephelometry to measure Lp(a) values (OR = 0.78; 95% CI: 0.63–0.98; *p* = 0.03; I^2^ = 87%), and those diagnosing MetS using HDM criteria (OR = 0.6; 95% CI: 0.39–0.93; *p* = 0.02; I^2^ = 90%) maintained a significant association with high heterogeneity. In the sensitivity analysis (Supplementary Figure S22), after eliminating studies at high risk of bias, the association was found to not remain (OR = 0.91; 95% CI: 0.76–1.1; *p* < 0.01, I^2^ = 93%).

### Publication bias

No asymmetry was found in the funnel plot (Supplementary Figures S23, 24, 25 and 26). No evidence of publication bias was found in any of the associations examined (Begg test > 0.1).

## Discussion

The primary objective of this systematic review and meta-analysis was to provide a comprehensive synthesis of the existing evidence regarding the relationship between ApoA1, ApoB, ApoB/A1 ratio and Lp(a), and MetS. The main findings of our investigation demonstrate evidence that high ApoB levels, low ApoA1 levels, and elevated ApoB/ApoA1 ratio are significantly associated with MetS. Despite the implementation of sensitivity analyses, our findings continue to demonstrate significant heterogeneity across the included studies.

Apolipoproteins are proteins synthesised in the liver that play a crucial role in the transport and redistribution of lipids [74, 75]. ApoA1, found in HDL, facilitates the reverse transport of peripheral cholesterol to the liver, thereby exerting an anti-atherogenic effect [74]. In contrast, ApoB, is responsible for transporting cholesterol to peripheral cells and may enhance atherothrombosis [75]. Thus, some studies have shown that increasing ApoA1 and decreasing ApoB have cardiovascular benefits, similar to our results. For example, the association between high ApoA1 levels and premature coronary heart disease is well known [76]. Additionally, individuals with smaller apolipoprotein A isoforms exhibit an approximately 2 times higher risk of developing coronary heart disease or experiencing ischaemic stroke than those with larger isoforms [9]. In contrast, a systematic review revealed that both statin and non-statin therapies effectively reduced cardiovascular risk by lowering ApoB levels [77]. Considering these findings, it is not surprising that the ratio of both lipoproteins has emerged as a cardiovascular marker. Thus, in the case of the ApoB/ApoA1 ratio, another systematic review demonstrated that elevated levels can enhance risk prediction of cardiovascular events, even after accounting for traditional risk factors, particularly in high-risk populations [78]. Although the explanation for the association between lipoprotein levels and cardiovascular risk is multifactorial [74, 75], it is plausible that some of these factors may also contribute to our observed associations between the levels of these markers and MetS.

The pathophysiology of MetS involves multiple mechanisms that are not yet not fully understood. There is ongoing debate regarding the individual components of MetS, in addition to genetic and epigenetic factors, represent distinct pathologies or are interconnected within a common broader pathogenic process [79]. Regardless of these mechanisms, they result in three major processes: hormonal activation, chronic inflammation and insulin resistance [79], and there is evidence of an association between apolipoprotein alterations and some of these mechanisms. In this regard, the well-established association between apolipoproteins, inflammation and insulin is widely recognised. For instance, ApoA1 has anti-inflammatory properties as evidenced by a study that identified 33 significant correlations between ApoA1 and urinary cytokine levels [80]. The strongest associations were observed for interleukin-1 alpha, spondin2, advanced glycation end-product receptor, protease-activated receptor-1, TNF-related apoptosis-inducing ligand receptor 2, interleukin-4 receptor alpha and stem cell factor [80]. Another study conducted on overweight and obese postmenopausal women showed that ApoB is the main predictor of inflammatory markers as it was an independent predictor of interindividual variation in IL-6, orosomucoid, haptoglobin and alpha 1-antitrypsin [81]. Regarding insulin resistance, numerous studies have established correlation between ApoA1, Apo B and ApoA1/ApoB ratio with insulin resistance in both diabetic and normo-glycemic patients [82–84]. However, the association between lipoproteins and insulin resistance appears to be a two-way relationship. This is due to the suggestion that under conditions of insulin resistance, the inhibitory effect of insulin on lipase activity is diminished, resulting in increased free fatty acids release through lipolysis. This, in turn, can lead to modifications in atherogenic lipoproteins, including the overproduction of ApoB [82].

It is worth emphasising that some authors consider the ApoB/ApoA1 ratio as the most accurate marker for assessing the balance between atherogenic and anti-atherogenic lipoproteins. They argue that this ratio serves as a superior predictor of cardiovascular risk associated with cardiovascular lipoproteins than traditional lipid indices [11, 85]. Similarly, it is hypothesised that it would be an ideal marker for lipid alterations associated with insulin resistance and MetS, as it captures the key characteristics of dyslipidemia associated with insulin resistance and MetS, including low HDL levels and elevated levels of VLDL and small dense LDL [11].

Despite the observed associations, the internal validity of our results is limited by their high heterogeneity, which can be attributed to inherent variations in the studies included in our study. Although sensitivity analysis was performed according to sex, diagnostic criteria for MetS, method of assessment of apolipoproteins and according to the continent in which the studies were conducted, the heterogeneity was still high, which means that other variables that can potentially affect these associations were not considered. One of these may be related to the prevalence of MetS, as it has been suggested that associations between ApoB and various cardiometabolic disorders are limited to populations with a relatively high prevalence of MetS [14]. Furthermore, it has been suggested that this association may not only vary based on the diagnosis of MetS but also by the number of diagnostic criteria utilised [83]. Similarly, this heterogeneity may be influenced by the cut-off point used to assess, for example, the ApoB/ApoA1 ratio, as some studies define it by numerical values and others by quartiles [50]. Likewise, although an elevated ApoB/ApoA1 ratio may imply high ApoB levels or low ApoA1 levels per se, its association with MetS may also reflect other factors associated with an elevated ApoB/ApoAI ratio that were not measured in the present study as suggested by its association with myocardial infarction [11]. This heterogeneity had a notable impact on the results concerning the association between Lp (a) and MetS, as after excluding studies with high risk of bias, the association was not maintained and high heterogeneity persisted in the remaining studies. Lp(a) primarily comprises LDL particles bound to apolipoprotein(a), is elevated in up to 20% of the general population and is associated with an elevated risk of atherothrombosis [86, 87]. Because mechanisms such as insulin resistance affect its concentration, it has been associated with the development of MetS, although the results of these associations remain controversial. Although our study does not resolve this controversy, it highlights the need for conducting more rigorous investigations to elucidate this association.

### Limitations and strenghts

Our study should be interpreted considering its limitations. First, it is important to note that the high heterogeneity observed between studies did not diminish even after conducting subgroup analysis and sensitivity analysis. This implies that there is high clinical and methodological variability among these studies, so it would be desirable that future studies take into account more variables that may influence the values of these lipoproteins (e.g. sociodemographic, comorbidities, and lifestyles). Second, due to the limited information in the studies, the sensitivity, specificity and optimal cut-off point of these markers for estimating the risk of developing MetS were not determined, which would be important to evaluate in future studies. Third, since the majority of the included studies were of cross-sectional design, there is a risk of reverse causality. Despite these limitations, our study has several strengths. A large number of studies were included, resulting in a substantial number of participants, thereby ensuring adequate statistical power. Furthermore, a thorough search was conducted across multiple databases, ensuring a comprehensive inclusion of the available evidence. Likewise, subgroup analysis was performed according to assay method, MetS diagnostic criteria, continents and study design. To the best of our knowledge, this systematic review and meta-analysis represents the first comprehensive synthesis of available studies examining the association between apolipoproteins and Lp(a) levels in patients with MetS.

## Conclusion

Increased values of ApoB and ApoB/ApoA1 ratio and reduced values of ApoA1 and Lp(a) are associated with the presence of MetS. These findings suggest that these lipid markers may serve as potential indicators for identifying subjects at risk of developing MetS. However, additional studies are warranted to gain a deeper understanding of the underlying mechanisms driving these associations. In addition, further clinical research and longitudinal studies are recommended to better understand the causal relationship between these lipid markers and MetS, as well as to explore their potential utility in clinical practice for early detection and management of MetS.

## Supplementary Information

**Additional file 1: Table S1.** Search strategies.** Table S2.** Criteria for clinical diagnosis of metabolic syndrome used in the included studies.** Table S3.** Quality assessment of included studies.** Figure S1.** Subgroup analysis according to assay method of the association between ApoB levels and MetS.** Figure S2.** Subgroup analysis according to diagnostic criteria of the association between ApoB levels and MetS.** Figure S3.** Subgroup analysis according to sex of the association between ApoB levels and MetS.** Figure S4.** Subgroup analysis according to continents of the association between ApoB levels and MetS.** Figure S5.** Subgroup analysis according to study design of the association between ApoB levels and MetS.** Figure S6.** Sensitivity analysis according to risk of bias of the association between ApoB levels and MetS.** Figure S7.** Subgroup analysis according to assay method of the association between ApoA1 levels and MetS.** Figure S8.** Subgroup analysis according to diagnostic criteria of the association between ApoA1 levels and MetS.** Figure S9.** Subgroup analysis according to sex of the association between ApoA1 levels and MetS.** Figure S10.** Subgroup analysis according to continents of the association between ApoA1 levels and MetS.** Figure S11.** Subgroup analysis according to study design of the association between ApoA1 levels and MetS.** Figure S12.** Sensitivity analysis according to risk of bias of the association between ApoA1 levels and MetS.** Figure S13.** Subgroup analysis according to diagnostic criteria of the association between ApoB/ApoA1 ratio levels and MetS.** Figure S14.** Subgroup analysis according to gender of the association between ApoB/ApoA1 ratio levels and MetS.** Figure S15.** Subgroup analysis according to continents of the association between ApoB/ApoA1 ratio levels and MetS.** Figure S16.** Subgroup analysis according to study design of the association between ApoB/ApoA1 ratio levels and MetS.** Figure S17.** Sensitivity analysis according to risk of bias of the association between ApoB/ApoA1 ratio levels and MetS.** Figure S18.** Subgroup analysis according to assay method of the association between lipoprotein(a) levels and MetS.** Figure S19.** Subgroup analysis according to diagnostic criteria of the association between lipoprotein(a) levels and MetS.** Figure S20.** Subgroup analysis according to continents of the association between lipoprotein(a) levels and MetS.** Figure S21.** Subgroup analysis according to study design of the association between lipoprotein(a) levels and MetS.** Figure S22.** Sensitivity analysis according to risk of bias of the association between lipoprotein(a) levels and MetS.** Figure S23.** Funnel Plot of the studies that evaluated the association between ApoB and MetS.** Figure S24.** Funnel Plot of the studies that evaluated the association between ApoA1 and MetS.** Figure S25.** Funnel Plot of the studies that evaluated the association between ApoB/ApoA1 ratio and MetS.** Figure S26.** Funnel Plot of the studies that evaluated the association between Lipoprotein(a) and MetS.

## Data Availability

All data generated or analysed during the current study are included in this published article and its supplementary information files.

## Abbreviations

ApoA1: Apolipoprotein A1
ApoB: Apolipoprotein B
Hcy: Homocysteine
LDL: Low Density Lipoprotein
Lp(a): Lipoprotein(a)
MetS: Metabolic syndrome
VLDL: Very Low-Density Lipoprotein
