# Supplementary material for: Association of apolipoproteins and lipoprotein(a) with metabolic syndrome: a systematic review and meta-analysis
Source: Lipids Health Dis. 2023 Jul 7;22:98. doi: 10.1186/s12944-023-01860-w (PMC10327137; doi:10.1186/s12944-023-01860-w)

**Association between Apolipoproteins and lipoprotein(a) values with metabolic syndrome: A systematic review and meta-analysis**

**Supplementary Material**

**Table S1. Search strategies**

| **Source** | **PubMed** |
| --- | --- |
| **Search** | **Formula** |
| **#1** | Metabolic Syndrome [MH] OR Cardiometabolic Syndrome [MH] OR Metabolic Syndrome X [MH] OR Reaven Syndrome X [MH] OR Insulin Resistance Syndrome X [MH] |
| **#2** | Apolipoproteins [MH] OR Lipoprotein(a) [MH] |
| **#3** | #1 AND #2 |
| **Source** | **Scopus** |
| **Search** | **Formula** |
| **#1** | TITLE-ABS-KEY(“Metabolic Syndrome*” OR “Cardiometabolic Syndrome*” OR “Metabolic Syndrome X*” OR “Insulin Resistance Syndrome X*” OR “Reaven Syndrome X*”) |
| **#2** | TITLE-ABS-KEY (“Apolipoproteins*”) |
| **#3** | TITLE-ABS-KEY (“Lipoprotein(a)*”) |
| **#4** | #2 OR #3 |
| **#5** | #1 AND #4 |
| **Source** | **Web of Science** |
| **Search** | **Formula** |
| **#1** | TI=(“Metabolic Syndrome*” OR “Cardiometabolic Syndrome*” OR “Metabolic Syndrome X*” OR “Insulin Resistance Syndrome X*” OR “Reaven Syndrome X*”) |
| **#2** | TI=(“Apolipoproteins*”) |
| **#3** | TI=(“Lipoprotein(a)*”) |
| **#4** | #2 OR #3 |
| **#5** | #1 AND #4 |
| **Source** | **Embase** |
| **Search** | **Formula** |
| **#1** | 'Metabolic Syndrome'/exp |
| **#2** | 'Apolipoproteins'/exp |
| **#3** | 'Lipoprotein(a)'/exp |
| **#4** | #2 OR #3 |
| **#5** | #1 AND #4 |
| **Source** | **OVID** |
| **Search** | **Formula** |
| **#1** | ('Metabolic Syndrome* OR Cardiometabolic Syndrome* OR Metabolic Syndrome X* OR Insulin Resistance Syndrome X* OR Reaven Syndrome X *).kw. |
| **#2** | (Apolipoproteins*).kw. |
| **#3** | ('Lipoprotein(a)*).kw. |
| **#4** | #2 OR #3 |
| **#5** | #1 AND #4 |

**Table S2. Criteria for clinical diagnosis of metabolic syndrome used in the included studies**

| **Diagnostic criteria** | **Definition** |
| --- | --- |
| **American Heart Association/National Heart Lung and Blood Institute (AHA/NHLBI)** | Three of more of following five criteria needed to be present: (1) waist circumference > 90 cm for men and >80 cm for women, (2) fasting serum triglycerides >= 150 mg/dl or on drug treatment for elevated triglycerides, (3) HDL cholesterol <40 mg/dl for men and <50 mg/dl for women or on drug treatment to increase HDL cholesterol, (4) fasting blood glucose >100 mg/dl or on drug treatment for diabetes and (5) systolic blood pressure >130 mmHg or diatolic > 85 mmHg or on drug treatment for hypertension. |
| **National Cholesterol Education Program-Adult Treatment Panel III(NCEP-ATP III)** | Three of the following factors: waist circumference >102cm (men) or >88cm(women), triglyceride level ≥1.69mmol/l, HDL cholesterol<1.03mmol/l (men) or < 1.29mmol/l (women), blood pressure ≥130/85mmHg, or fasting glucose ≥6.1mmol/l. |
| **International Diabetes Federation(IDF)** | Central obesity plus any two of the following four factors: raised fasting plasma glucose (≥100 mg/dL) or treatment of previously diagnosed type 2 diabetes, raised blood pressure (systolic blood pressure ≥130 or diastolic blood pressure ≥85 mmHg) or treatment of previously diagnosed hypertension, raised triglycerides (≥150 mg/dl), and reduced HDL cholesterol (<40 mg/dL in males, <50 mg/dL in females). |
| **Chinese Diabetes Society(CDS)** | Three of the following 5 criteria: abdominal obesity (waistline ≥90 cm in adult male and 85 cm in adult female subjects), elevated blood pressure (SBP ≥130 mmHg and/or DBP ≥85 mmHg, or on treatment for diagnosed hypertension formerly), hyperglycemia (FBG ≥6.1 mmol/L, or on appropriate hypoglycemic medication), elevated TG (≥1.7 mmol/L, or on appropriate lipid-lowering medication) and reduced HDL-C (<1.03 mmol/L in adult male and <1.30 mmol/L in adult female subjects, or on appropriate lipid-lowering medication). |
| **World Health Organization(WHO)** | Impaired glucose intolerance, Impaired fasting glycemiaI, T2DM, or lowered insulin sensitivity plus any 2 of the following: (1)Men: waist-to-hip ratio >0.90,women: waist-to-hip ratio >0.85 and/or BMI >30; (2) TG ≥150 mg/dL and/or HDL-C <35 mg/dL in men or <39 mg/dL in women; systolic blood pressure ≥140 mmHg or diatolic ≥ 90 mmHg and (3) microalbuminuria |
| **Harmonized Definition of MetS (HDM)** | Three or more of the following: 1) waist circumference ≥ 90 cm in menand ≥85 cm in women; 2) triglycerides ≥150 mg/dL; 3) HDL cholesterol <40 mg/dL inmen and <50 mg/dL in women; 4) blood pressure ≥ 130/85 mm Hg or on antihypertensivemedication; and 5) fasting glucose ≥ 100 mg/dL or on antidiabetic medication. |

**Table S3. Quality assessment of included studies**

|  | **NEWCASTLE - OTTAWA QUALITY ASSESSMENT SCALE FOR COHORT STUDIES** | | | | | | | | | | |
| --- | --- | --- | --- | --- | --- | --- | --- | --- | --- | --- | --- |
| **STUDY** | | **SELECTION** | | | | **COMPARABILITY** | **OUTCOME** | |  |  |  |
|  | | **Representativeness of the exposed cohort** | **Selection of the non-exposed cohort** | **Ascertainment of exposure** | **Demonstration that outcome of interest was not present at start of study** | **Comparability of Cohorts on the Basis of the Design or Analysis Maximum : ☆☆** | **Assessment of outcome** | **Was follow-up long enough for outcomes to occur** | **Adequacy of follow up of cohorts** | **SCORE** | **Evidence quality** |
| **Chou YL et al. (A)** | | ☆ | ☆ | ☆ | ☆ | ☆☆ | ☆ | ☆ | ☆ | 9 | Low risk of bias |
| **Chou YL et al. (B)** | | ☆ | ☆ | ☆ | ☆ | ☆ | ☆ | ☆ | ☆ | 8 | Low risk of bias |
| **Sung K et al.** | | ☆ | ☆ | ☆ | ☆ | ☆☆ | ☆ | ☆ | ☆ | 9 | Low risk of bias |
| **Borja M et al.** | | ☆ | ☆ | ☆ | ☆ | ☆ | ☆ | ☆ | ☆ | 8 | Low risk of bias |

|  | |  | | **NEWCASTLE - OTTAWA QUALITY ASSESSMENT SCALE FOR CASE-CONTROL STUDIES** | | | | | | | | | |
| --- | --- | --- | --- | --- | --- | --- | --- | --- | --- | --- | --- | --- | --- |
| **STUDY** | | **SELECTION** | | | | **COMPARABILITY** | | **EXPOSURE** | |  |  |  |  |
|  | | **Is the case definition adequate?** | | **Representativeness of the cases** | **Selection of Controls** | **Definition of Controls** | **Comparability of cases and controls on the basis of the design or analysis (Maximum : ☆☆ )** |  | **Same method of ascertainment for cases and controls** | **Non-Response rate** | **Ascertainment of exposure** | **SCORE** | **Evidence quality** |
| **Savinova O et al.** | | ☆ | | ☆ | ☆ | ☆ | ☆ | | ☆ | ☆ | ☆ | 8 | Low risk of bias |
| **De Souza J et al.** | | ☆ | | ☆ | ☆ | ☆ | ☆☆ | | ☆ | ☆ | ☆ | 9 | Low risk of bias |
| **Belfki H et al.** | | ☆ | | ☆ | ☆ | ☆ | ☆ | | ☆ | ☆ | ☆ | 8 | Low risk of bias |
| **Barkas F et al.** | | ☆ | | ☆ | ☆ | ☆ | ☆ | | ☆ | ☆ | ☆ | 8 | Low risk of bias |
| **Reynoso-Villalpando G et al.** | | ☆ | | ☆ |  |  | ☆ | | ☆ | ☆ | ☆ | 6 | High risk of bias |
| **Boumaiza I et al.** | | ☆ | | ☆ | ☆ | ☆ | ☆☆ | | ☆ | ☆ | ☆ | 9 | Low risk of bias |
| **Sierra-Johnson J et al.** | | ☆ | | ☆ | ☆ | ☆ | ☆☆ | | ☆ | ☆ | ☆ | 9 | Low risk of bias |

|  | **NEWCASTLE - OTTAWA QUALITY ASSESSMENT SCALE FOR CROSS-SECTIONAL STUDIES** | | | | | | | | | |
| --- | --- | --- | --- | --- | --- | --- | --- | --- | --- | --- |
| **STUDY** | | ***SELECTION*** | | | | ***COMPARABILITY*** | ***OUTCOME*** | |  |  |
|  | | ***Representativeness of the sample*** | ***Sample size*** | ***Non-respondents*** | ***Ascertainment of the exposure (risk factor)*** | ***The subjects in different outcome groups are comparable, based on the study design or analysis. Confounding factors are controlled.***  ***Maximum : ☆☆*** | ***Assessment of outcome*** | ***Statistical test*** | ***SCORE*** | ***Evidence quality*** |
| **Lind L et al.** | | ☆ | ☆ | ☆ | ☆ | ☆☆ | ☆ | ☆ | 8 | Low Risk of Bias |
| **Won S et al. (A)** | | ☆ | ☆ | ☆ | ☆ | ☆ | ☆ |  | 6 | High Risk of Bias |
| **Won S et al. (B)** | | ☆ | ☆ | ☆ | ☆ | ☆ | ☆ |  | 6 | High Risk of Bias |
| **Pitsavos C et al.** | | ☆ | ☆ | ☆ | ☆ | ☆ | ☆ |  | 6 | High Risk of Bias |
| **Hee C et al. (A)** | | ☆ | ☆ | ☆ | ☆ | ☆☆ | ☆ | ☆ | 8 | Low Risk of Bias |
| **Hee C et al. (B)** | | ☆ | ☆ | ☆ | ☆ | ☆☆ | ☆ | ☆ | 8 | Low Risk of Bias |
| **Lim Y et al.** | | ☆ | ☆ | ☆ | ☆ | ☆☆ | ☆ | ☆ | 8 | Low Risk of Bias |
| **Dullaart R et al.** | | ☆ | ☆ | ☆ | ☆ | ☆☆ | ☆ | ☆ | 8 | Low Risk of Bias |
| **Sreckovic B et al.** | | ☆ | ☆ | ☆ | ☆ | ☆☆ | ☆ | ☆ | 8 | Low Risk of Bias |
| **Mattsson N et al.** | | ☆ | ☆ | ☆ | ☆ | ☆☆ | ☆ | ☆ | 8 | Low Risk of Bias |
| **Nurtazina A et al.** | | ☆ | ☆ | ☆ | ☆ | ☆☆ | ☆ | ☆ | 8 | Low Risk of Bias |
| **Won D et al.** | | ☆ | ☆ | ☆ | ☆ | ☆☆ | ☆ | ☆ | 8 | Low Risk of Bias |
| **Du R et al.** | | ☆ | ☆ | ☆ | ☆ | ☆☆ | ☆ | ☆ | 8 | Low Risk of Bias |
| **Hye J et al. (A)** | | ☆ | ☆ | ☆ | ☆ | ☆☆ | ☆ | ☆ | 8 | Low Risk of Bias |
| **Hye J et al. (B)** | | ☆ | ☆ | ☆ | ☆ | ☆☆ | ☆ | ☆ | 8 | Low Risk of Bias |
| **Pei W et al.** | | ☆ | ☆ | ☆ | ☆ | ☆☆ | ☆ | ☆ | 8 | Low Risk of Bias |
| **Bonora E et al.** | | ☆ | ☆ | ☆ | ☆ | ☆☆ | ☆ | ☆ | 8 | Low Risk of Bias |
| **Cankurtaran M et al.** | | ☆ | ☆ | ☆ | ☆ | ☆ | ☆ |  | 6 | High Risk of Bias |
| **Muntner P et al.** | | ☆ | ☆ | ☆ | ☆ | ☆☆ | ☆ | ☆ | 8 | Low Risk of Bias |
| **Panagiotakos D et al.** | | ☆ | ☆ | ☆ | ☆ | ☆☆ | ☆ | ☆ | 8 | Low Risk of Bias |
| **Rohit A et al.** | | ☆ | ☆ | ☆ | ☆ | ☆☆ | ☆ | ☆ | 8 | Low Risk of Bias |
| **Li Y et al.** | | ☆ | ☆ | ☆ | ☆ | ☆☆ | ☆ | ☆ | 8 | Low Risk of Bias |
| **Andrea G et al.** | | ☆ | ☆ | ☆ | ☆ | ☆☆ | ☆ | ☆ | 8 | Low Risk of Bias |
| **Sharan H et al.** | | ☆ | ☆ | ☆ | ☆ | ☆☆ | ☆ | ☆ | 8 | Low Risk of Bias |
| **Blatter M et al.** | | ☆ | ☆ | ☆ | ☆ | ☆☆ | ☆ | ☆ | 8 | Low Risk of Bias |
| **Jing F et al.** | | ☆ | ☆ | ☆ | ☆ | ☆☆ | ☆ | ☆ | 8 | Low Risk of Bias |
| **He H et al.** | | ☆ | ☆ | ☆ | ☆ | ☆☆ | ☆ | ☆ | 8 | Low Risk of Bias |
| **Park J et al.** | | ☆ | ☆ | ☆ | ☆ | ☆☆ | ☆ | ☆ | 8 | Low Risk of Bias |
| **Makaridze Z et al. (A)** | | ☆ | ☆ | ☆ | ☆ | ☆☆ | ☆ | ☆ | 8 | Low Risk of Bias |
| **Makaridze Z et al. (B)** | | ☆ | ☆ | ☆ | ☆ | ☆☆ | ☆ | ☆ | 8 | Low Risk of Bias |
| **Boiko A et al.** | | ☆ | ☆ | ☆ | ☆ | ☆☆ | ☆ | ☆ | 8 | Low Risk of Bias |
| **Al-Daghri N et al.** | | ☆ | ☆ | ☆ | ☆ | ☆ | ☆ |  | 6 | High Risk of Bias |
| **Wang W et al.** | | ☆ | ☆ | ☆ | ☆ | ☆☆ | ☆ | ☆ | 8 | Low Risk of Bias |
| **Guven A et al.** | | ☆ | ☆ | ☆ | ☆ | ☆☆ | ☆ | ☆ | 8 | Low Risk of Bias |
| **Wu X et al.** | | ☆ | ☆ | ☆ | ☆ | ☆☆ | ☆ | ☆ | 8 | Low Risk of Bias |
| **Cardoso-Saldaña G et al.** | | ☆ | ☆ | ☆ | ☆ | ☆ | ☆ |  | 6 | High Risk of Bias |
| **Jun J et al.** | | ☆ | ☆ | ☆ | ☆ | ☆☆ | ☆ | ☆ | 8 | Low Risk of Bias |
| **Onat A et al.** | | ☆ | ☆ | ☆ | ☆ | ☆ | ☆ |  | 6 | High Risk of Bias |
| **Gentile M et al.** | | ☆ | ☆ | ☆ | ☆ | ☆☆ | ☆ | ☆ | 8 | Low Risk of Bias |
| **Vaverková H et al.** | | ☆ | ☆ | ☆ | ☆ | ☆ | ☆ |  | 6 | High Risk of Bias |
| **Prasad M et al.** | | ☆ | ☆ | ☆ | ☆ | ☆ | ☆ |  | 6 | High Risk of Bias |
| **Kotani K et al.** | | ☆ | ☆ | ☆ | ☆ | ☆☆ | ☆ | ☆ | 8 | Low Risk of Bias |
| **Mokhsin A et al. (A)** | | ☆ | ☆ | ☆ | ☆ | ☆☆ | ☆ | ☆ | 8 | Low Risk of Bias |
| **Mokhsin A et al. (B)** | | ☆ | ☆ | ☆ | ☆ | ☆☆ | ☆ | ☆ | 8 | Low Risk of Bias |
| **Riediger N et al.** | | ☆ | ☆ | ☆ | ☆ | ☆☆ | ☆ | ☆ | 8 | Low Risk of Bias |

**Figure S1. Subgroup analysis according to assay method of the association between ApoB levels and MetS**

**
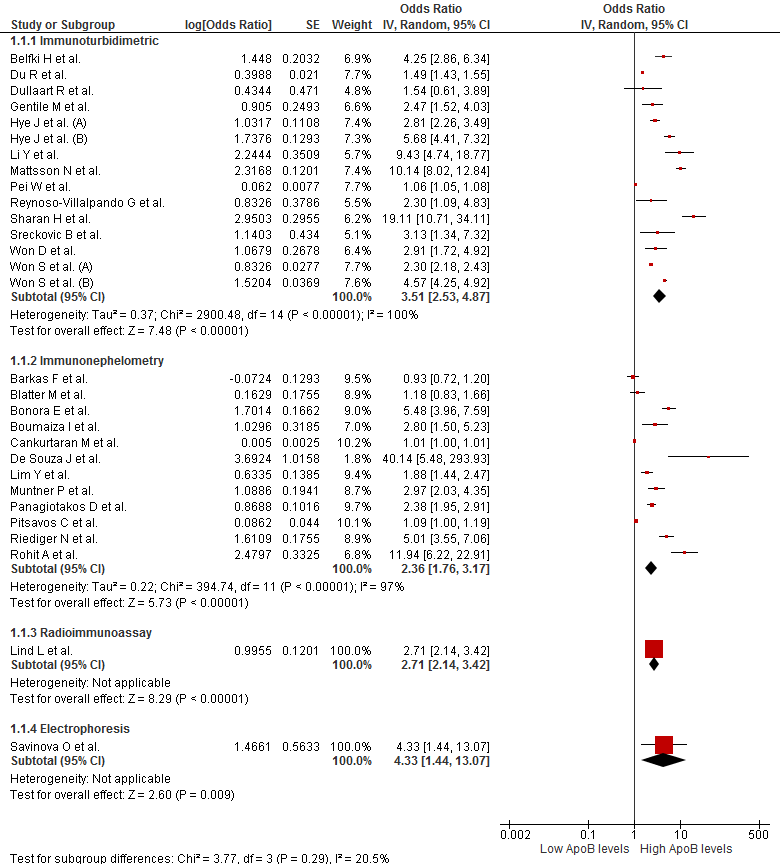
**

**Figure S2. Subgroup analysis according to diagnostic criteria of the association between ApoB levels and MetS**

**
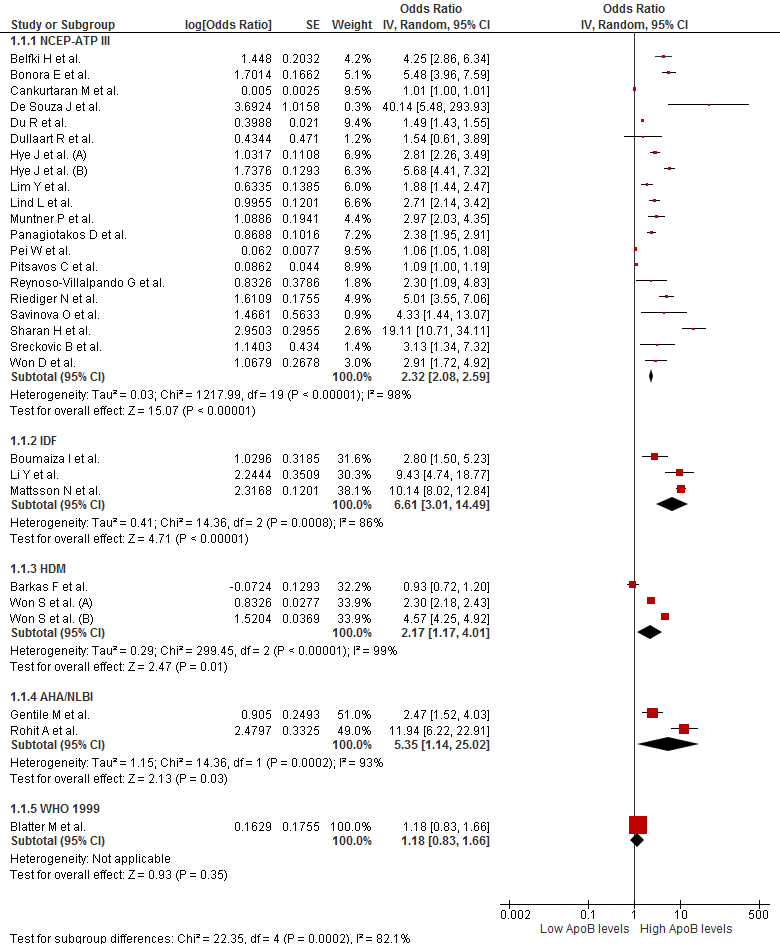
**

**Figure S3. Subgroup analysis according to sex of the association between ApoB levels and MetS**

**
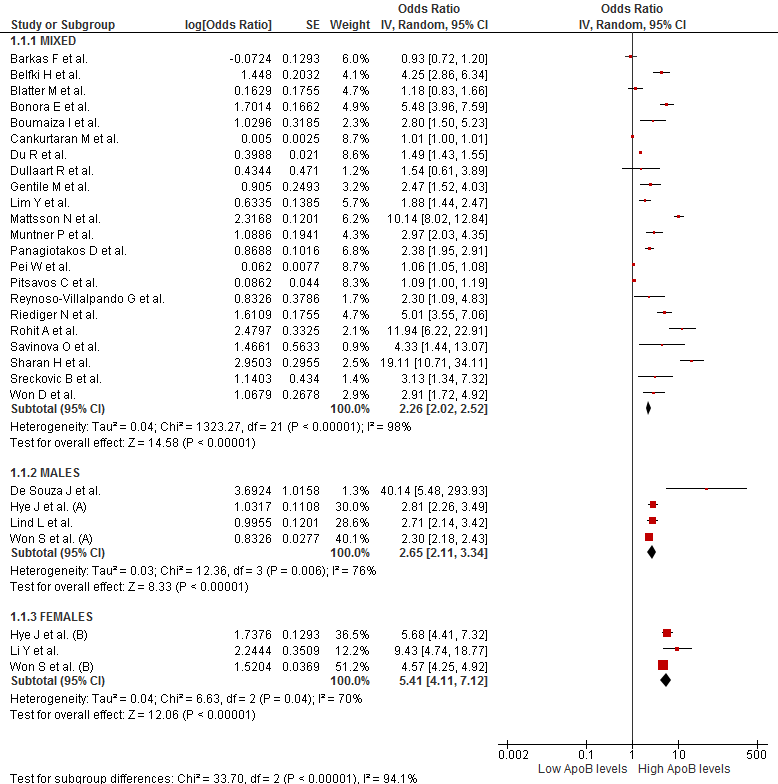
**

**Figure S4. Subgroup analysis according to continents of the association between ApoB levels and MetS**

**
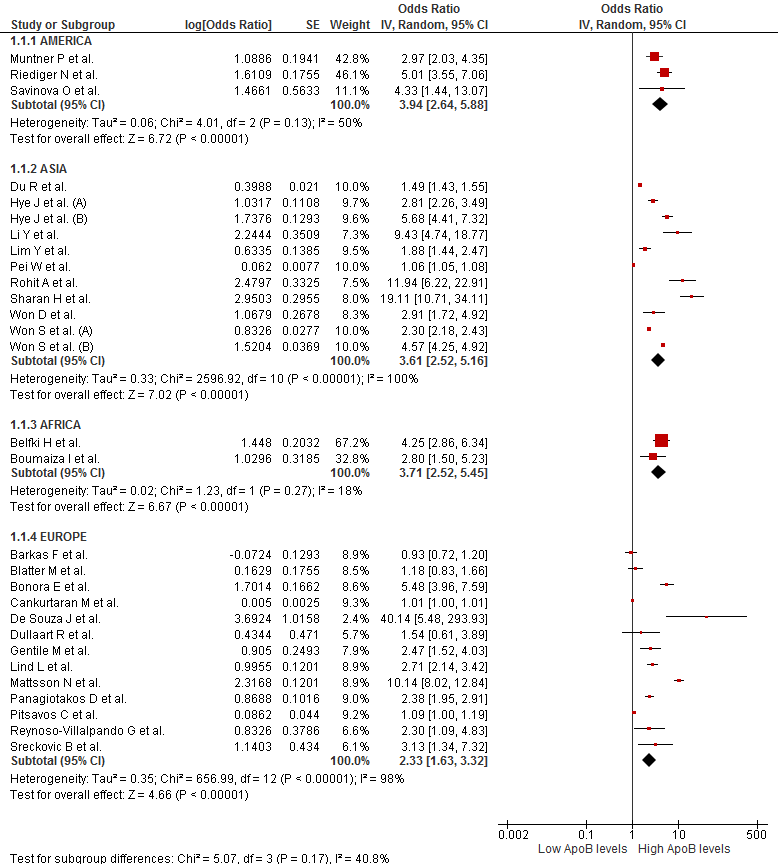
**

**Figure S5. Subgroup analysis according to study design of the association between ApoB levels and MetS**

**
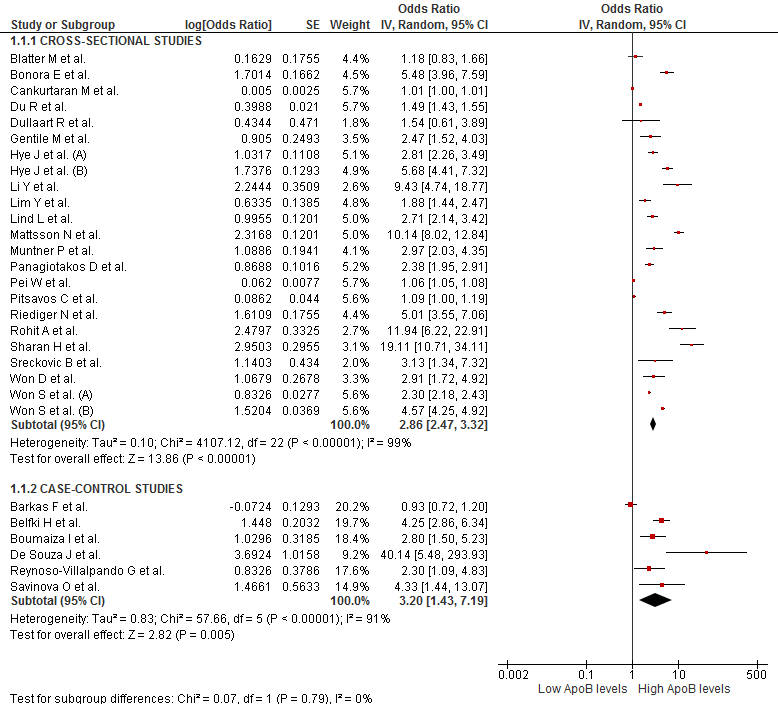
**

**Figure S6. Sensitivity analysis according to risk of bias of the association between ApoB levels and MetS**

**
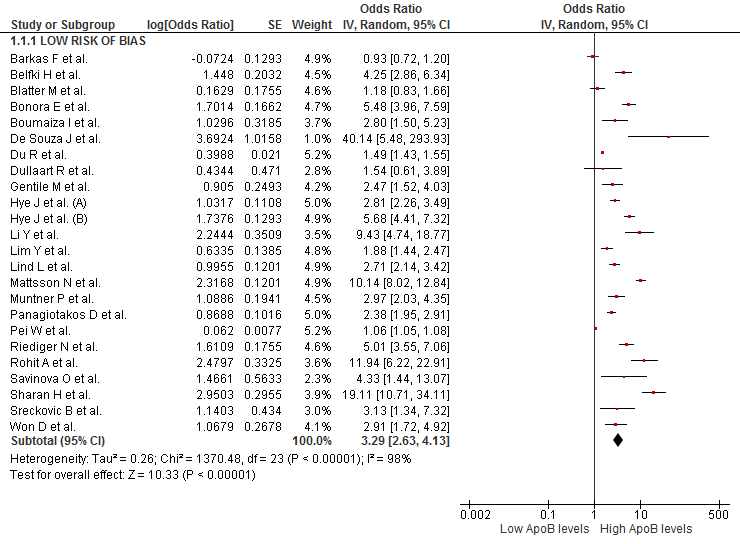
**

**Figure S7. Subgroup analysis according to assay method of the association between ApoA1 levels and MetS**


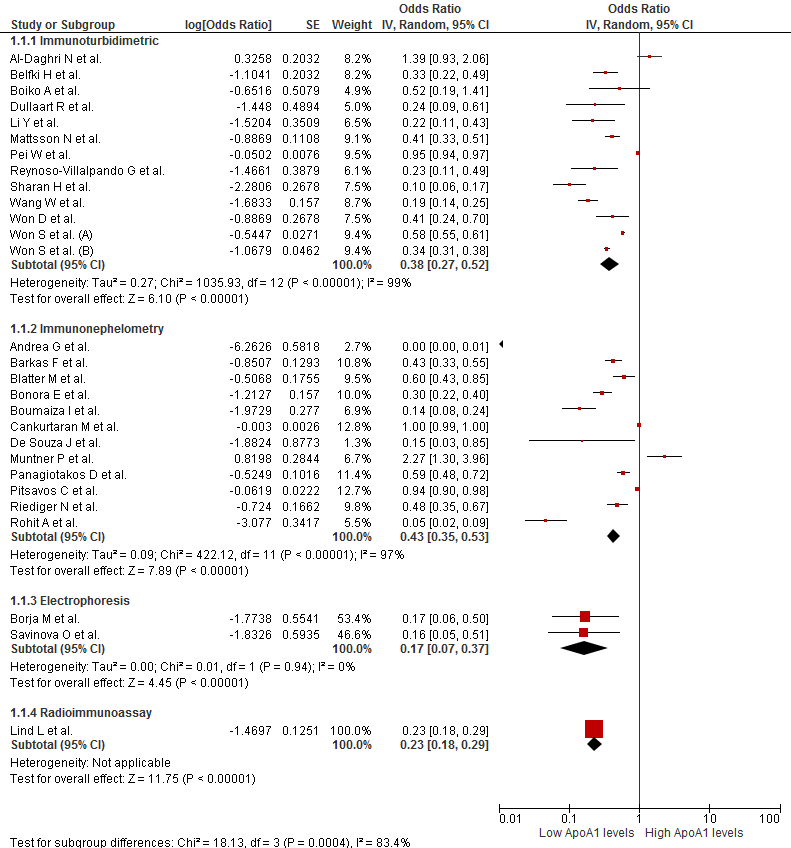


**Figure S8. Subgroup analysis according to diagnostic criteria of the association between ApoA1 levels and MetS**

**
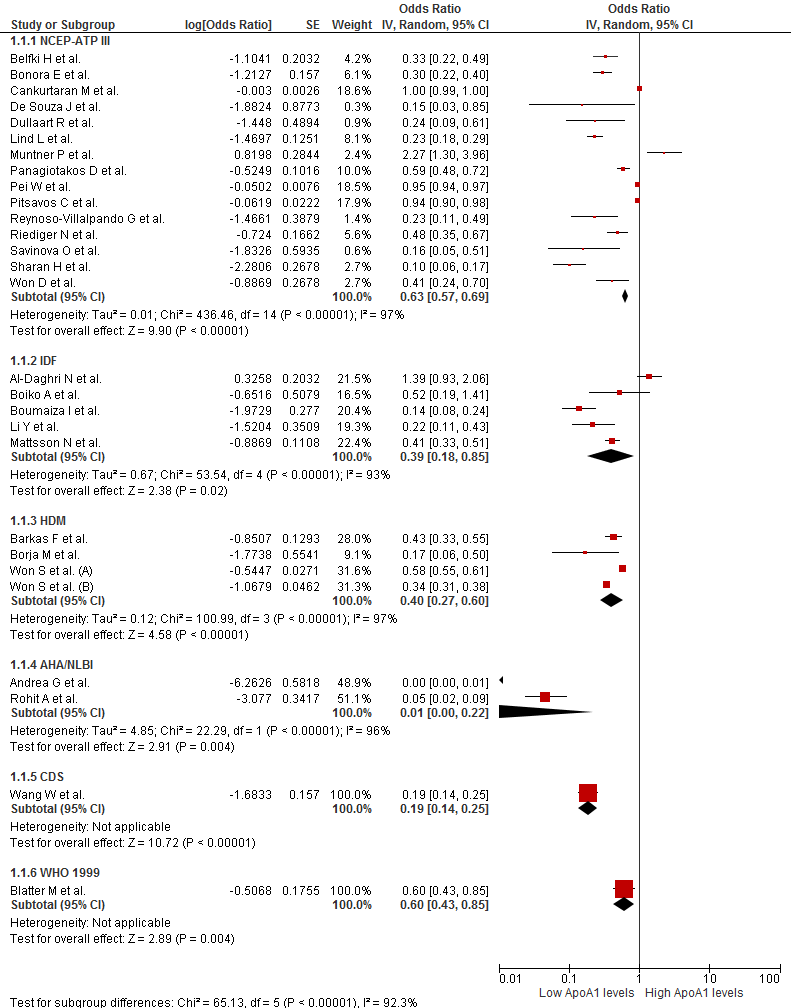
**

**Figure S9. Subgroup analysis according to sex of the association between ApoA1 levels and MetS**

**
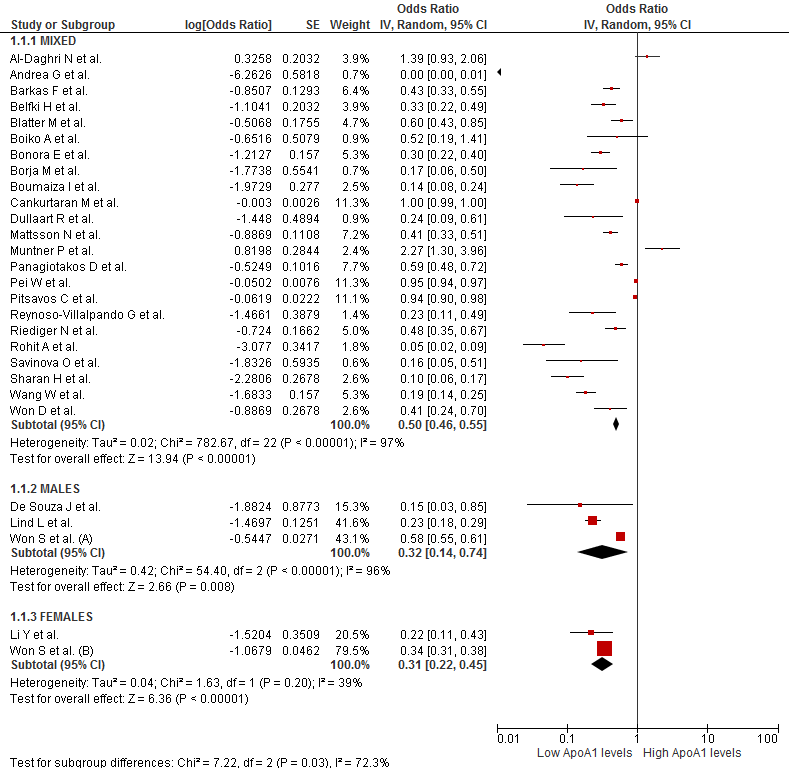
**

**Figure S10. Subgroup analysis according to continents of the association between ApoA1 levels and MetS**


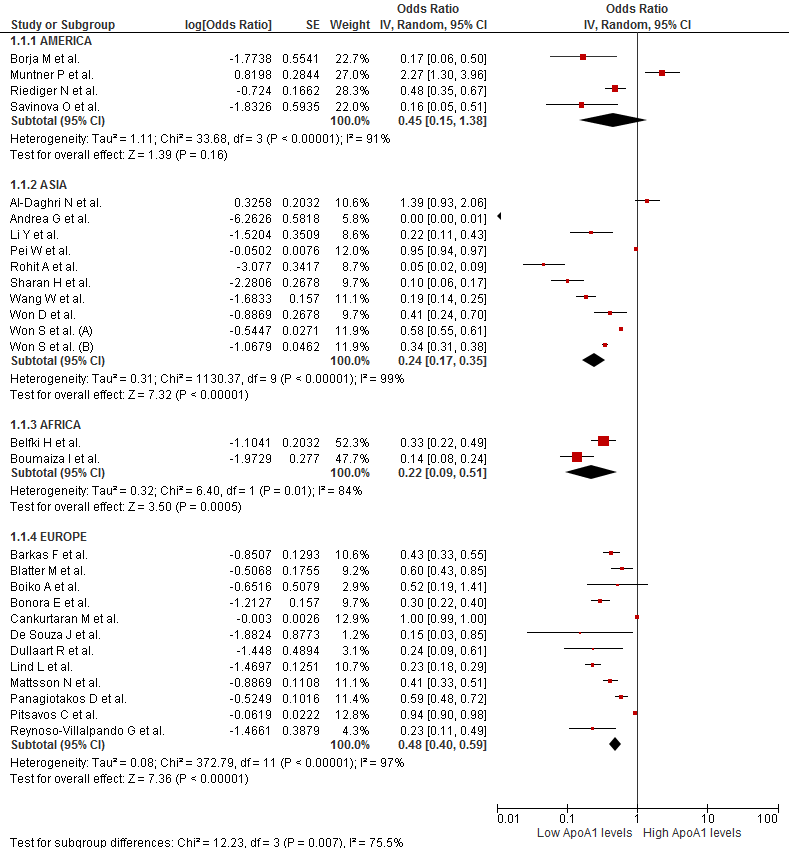


**Figure S11. Subgroup analysis according to study design of the association between ApoA1 levels and MetS**

**
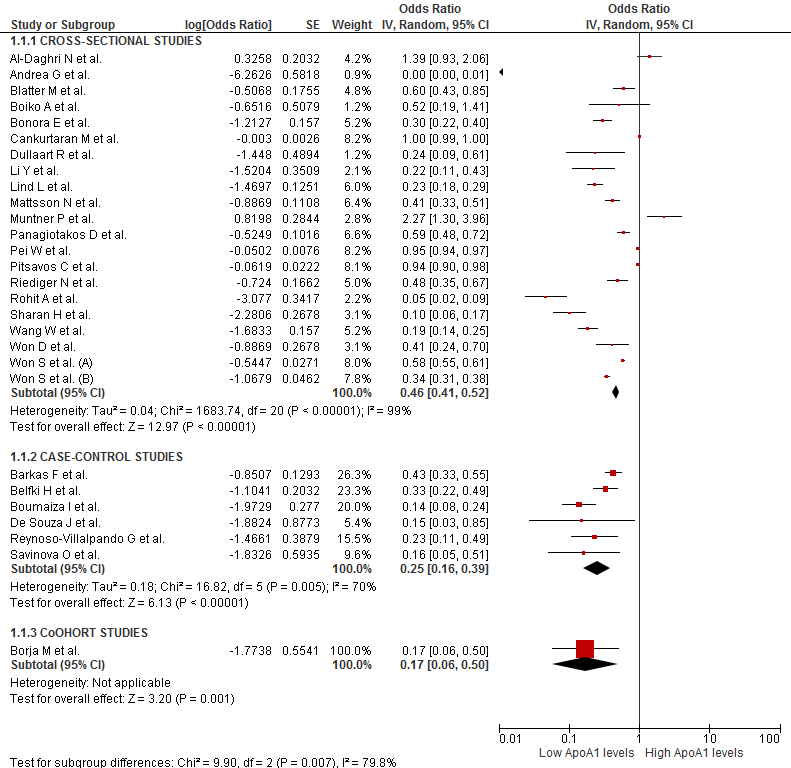
**

**Figure S12. Sensitivity analysis according to risk of bias of the association between ApoA1 levels and MetS**

**
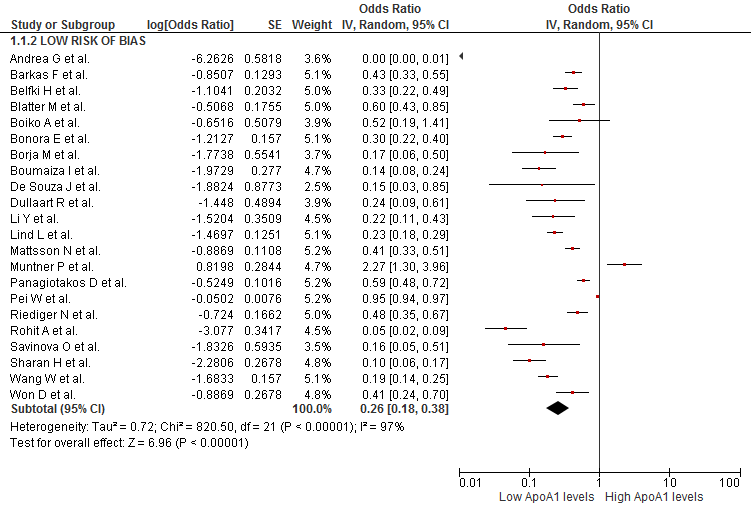
**

**Figure S13. Subgroup analysis according to diagnostic criteria of the association between ApoB/ApoA1 ratio levels and MetS**

**
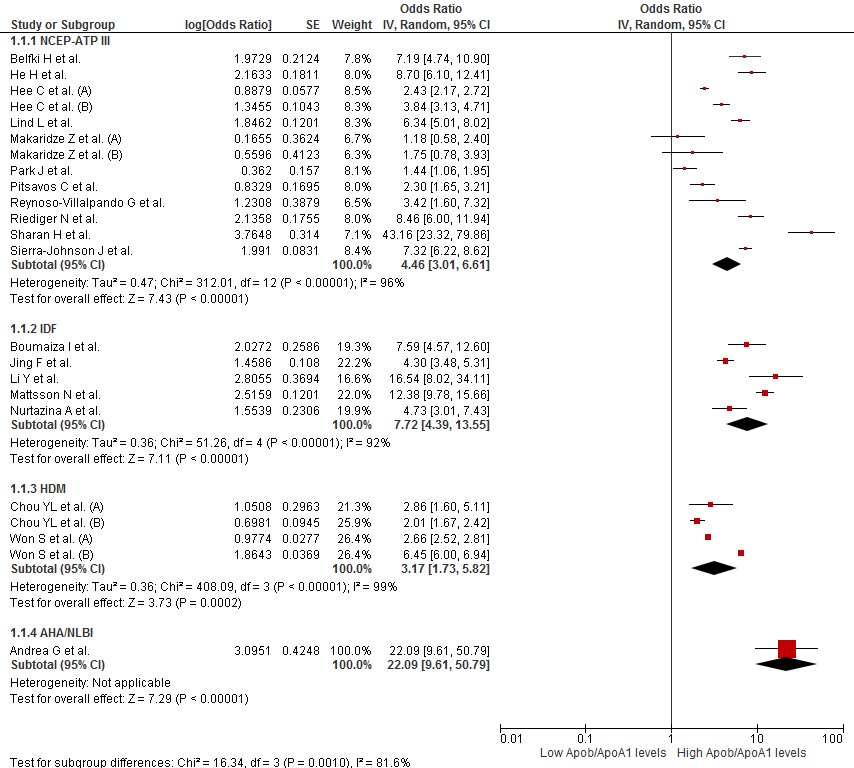
**

**Figure S14. Subgroup analysis according to gender of the association between ApoB/ApoA1 ratio levels and MetS**

**
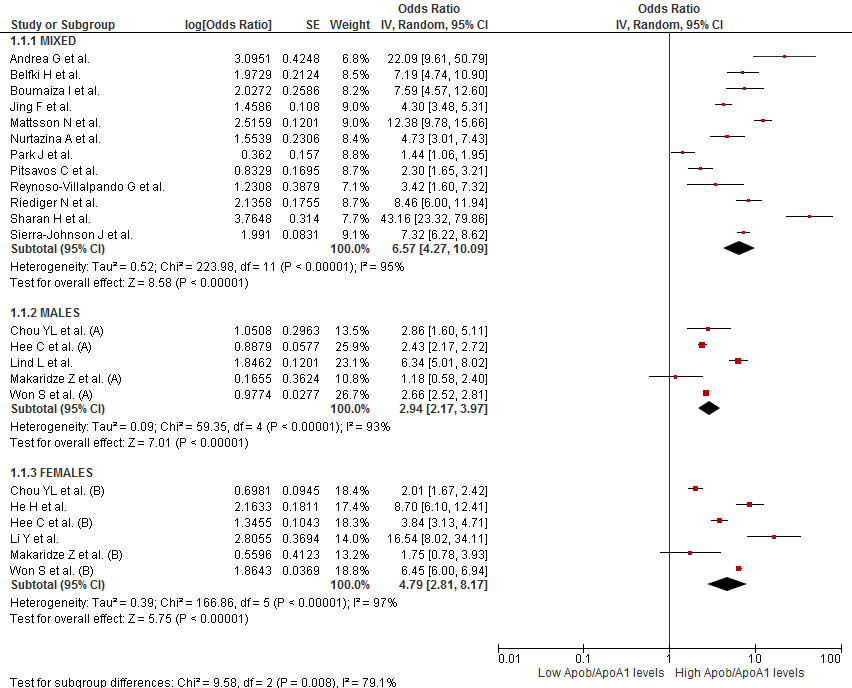
**

**Figure S15. Subgroup analysis according to continents of the association between ApoB/ApoA1 ratio levels and MetS**


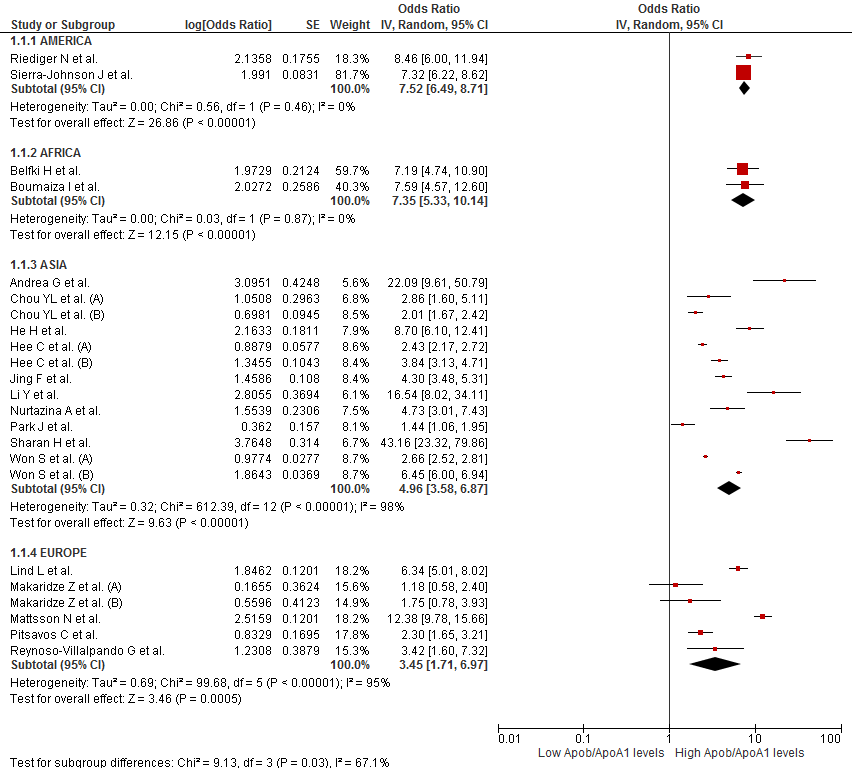


**Figure S16. Subgroup analysis according to study design of the association between ApoB/ApoA1 ratio levels and MetS**

**
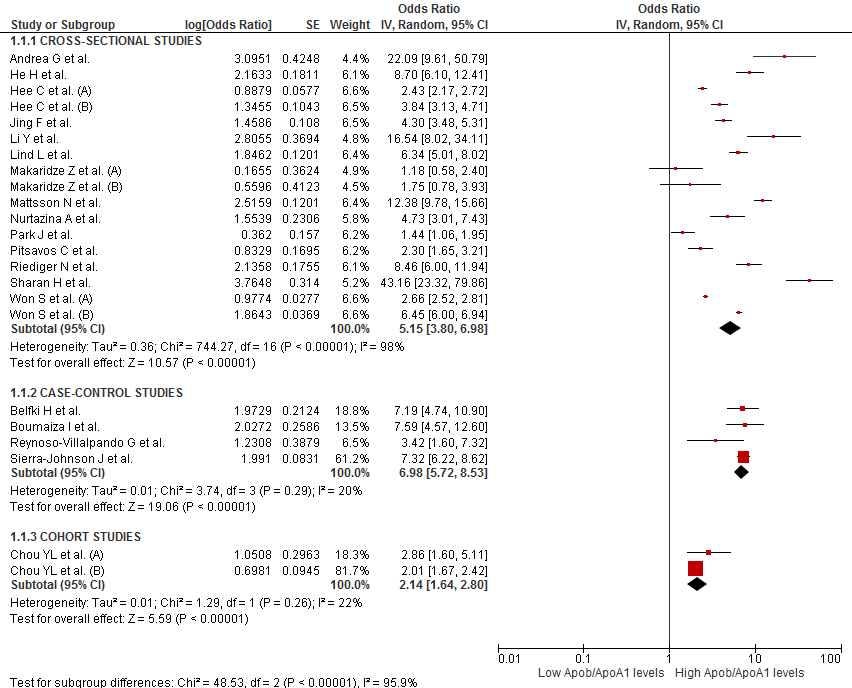
**

**Figure S17. Sensitivity analysis according to risk of bias of the association between ApoB/ApoA1 ratio levels and MetS**


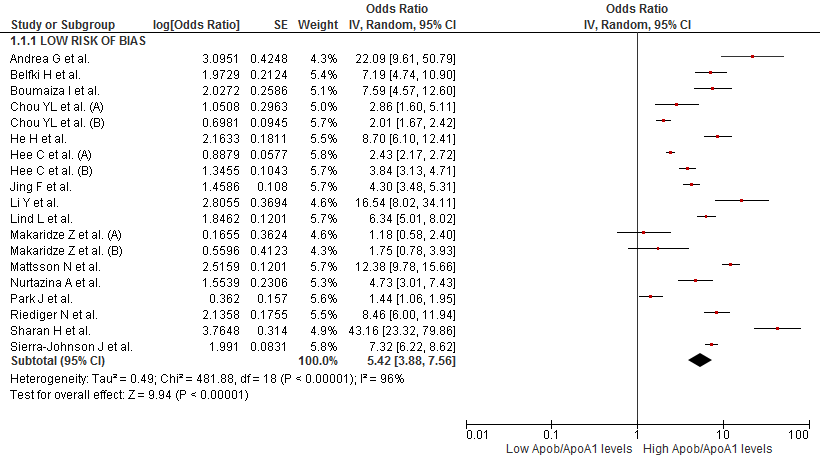


**Figure S18. Subgroup analysis according to assay method of the association between lipoprotein(a) levels and MetS**


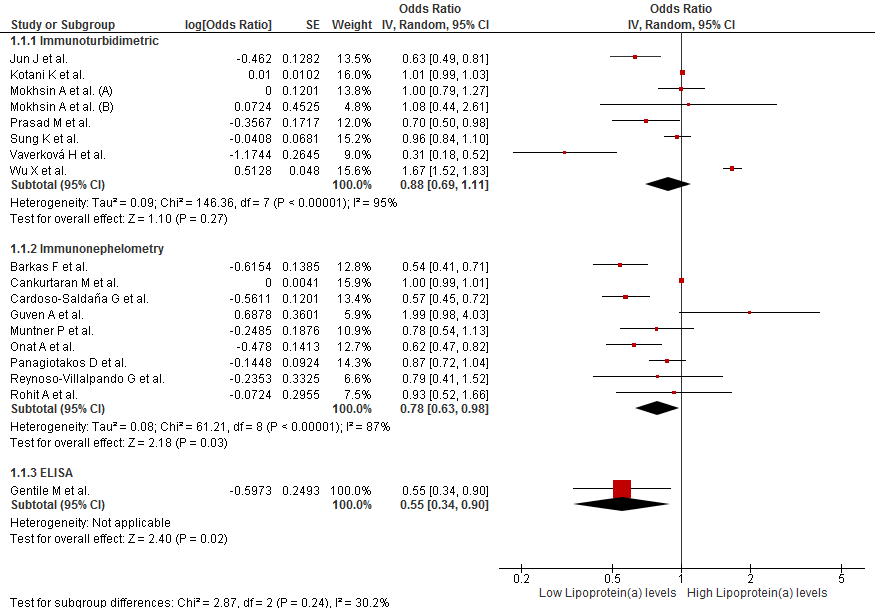


**Figure S19. Subgroup analysis according to diagnostic criteria of the association between lipoprotein(a) levels and MetS**

**
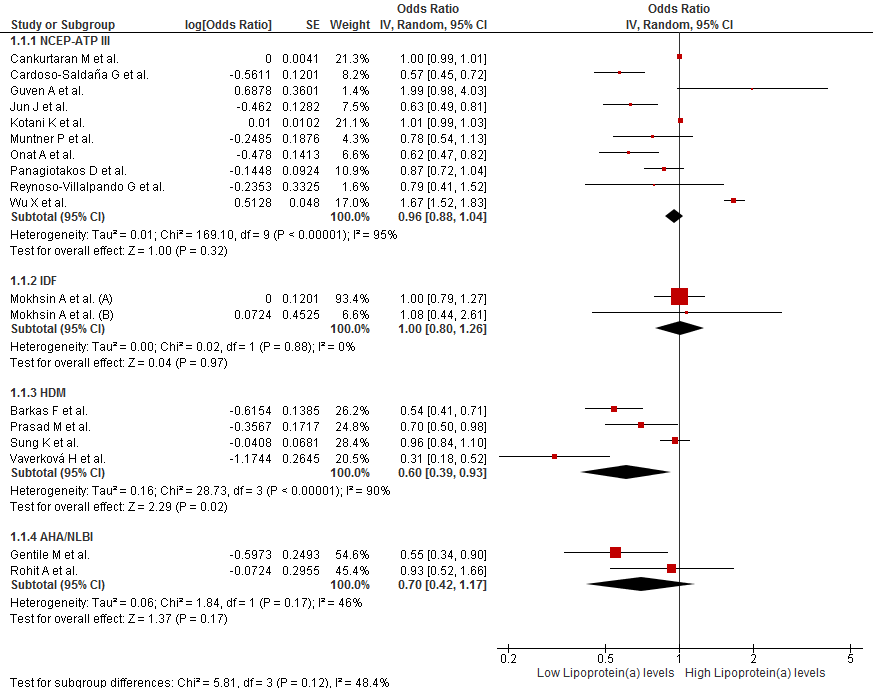
**

**Figure S20. Subgroup analysis according to continents of the association between lipoprotein(a) levels and MetS**


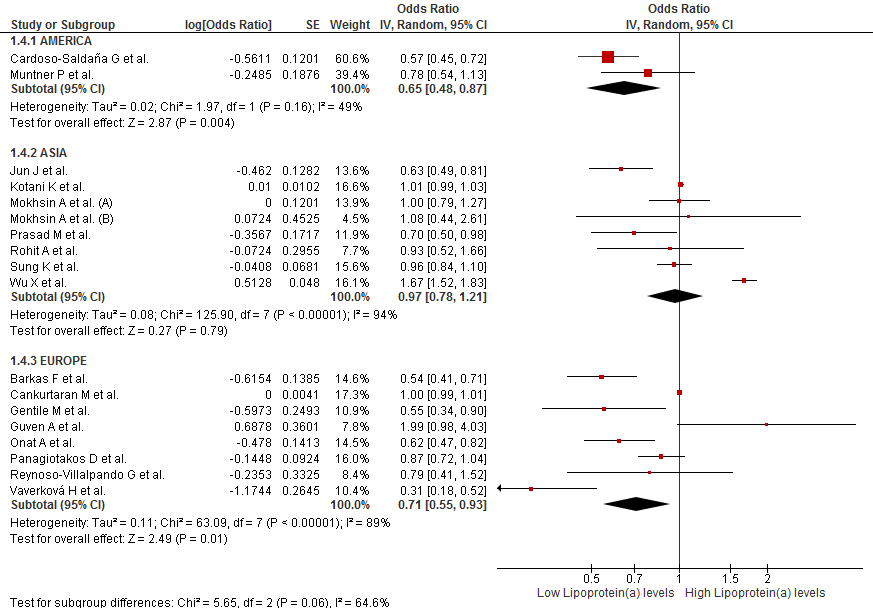


**Figure S21. Subgroup analysis according to study design of the association between lipoprotein(a) levels and MetS**


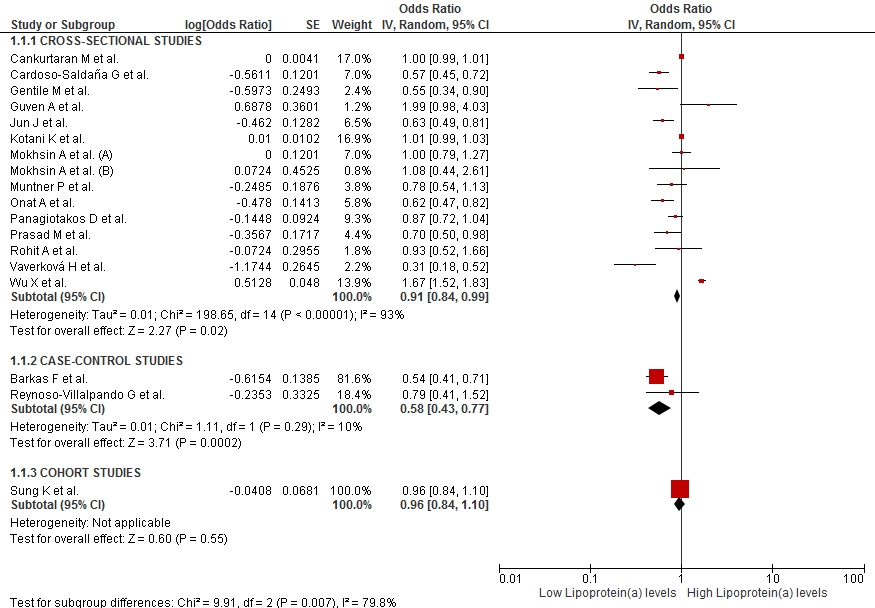


**Figure S22. Sensitivity analysis according to risk of bias of the association between lipoprotein(a) levels and MetS**


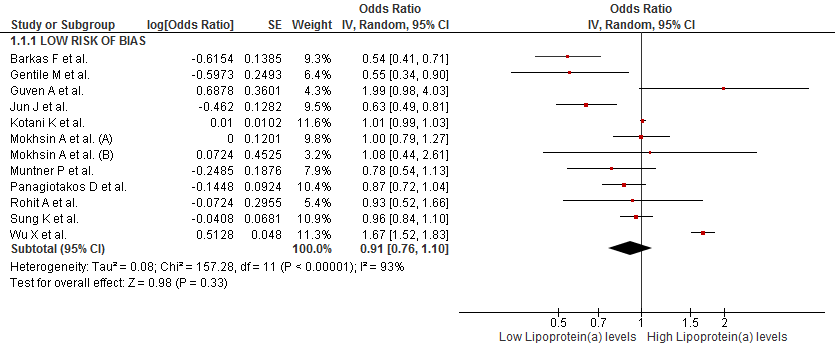


**Figure S23. Funnel Plot of the studies that evaluated the association between ApoB and MetS**


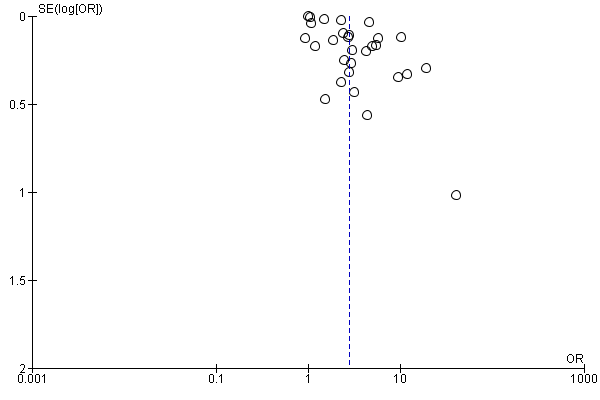


**Figure S24. Funnel Plot of the studies that evaluated the association between ApoA1 and MetS**


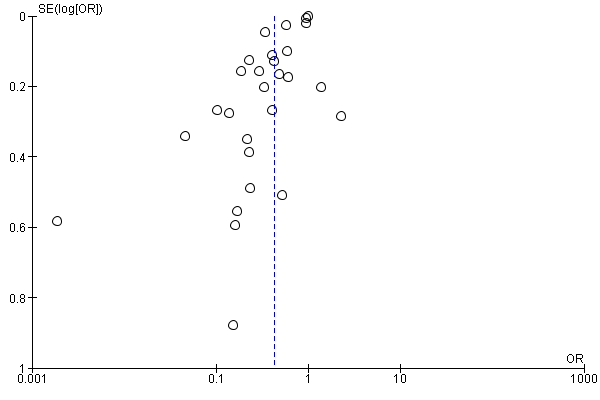


**Figure S25. Funnel Plot of the studies that evaluated the association between ApoB/ApoA1 ratio and MetS**


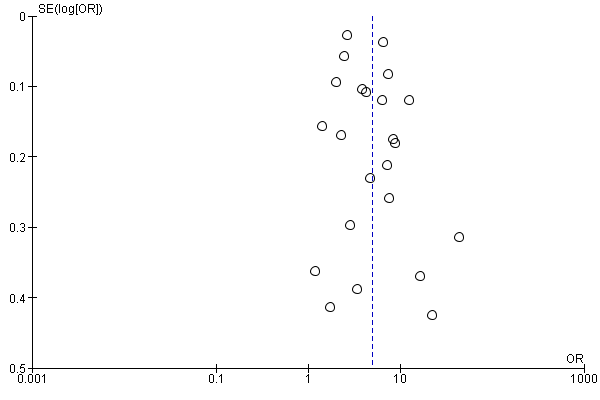


**Figure S26. Funnel Plot of the studies that evaluated the association between Lipoprotein (a) and MetS**


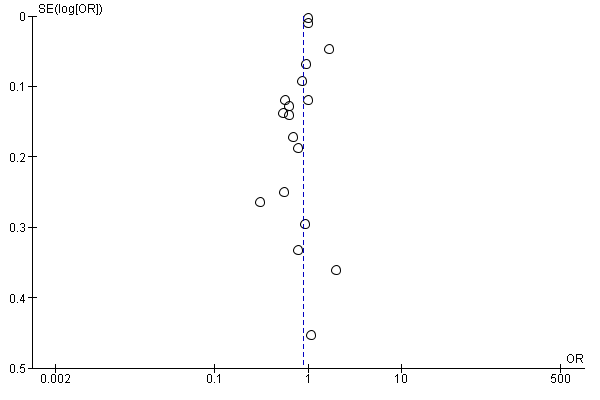

Supplement: Supplementary file 1 — Additional file 1: Table S1. Search strategies. Table S2. Criteria for clinical diagnosis of metabolic syndrome used in the included studies. Table S3. Quality assessment of included studies. Figure S1. Subgroup analysis according to assay method of the association between ApoB levels and MetS. Figure S2. Subgroup analysis according to diagnostic criteria of the association between ApoB levels and MetS. Figure S3. Subgroup analysis according to sex of the association between ApoB levels and MetS. Figure S4. Subgroup analysis according to continents of the association between ApoB levels and MetS. Figure S5. Subgroup analysis according to study design of the association between ApoB levels and MetS. Figure S6. Sensitivity analysis according to risk of bias of the association between ApoB levels and MetS. Figure S7. Subgroup analysis according to assay method of the association between ApoA1 levels and MetS. Figure S8. Subgroup analysis according to diagnostic criteria of the association between ApoA1 levels and MetS. Figure S9. Subgroup analysis according to sex of the association between ApoA1 levels and MetS. Figure S10. Subgroup analysis according to continents of the association between ApoA1 levels and MetS. Figure S11. Subgroup analysis according to study design of the association between ApoA1 levels and MetS. Figure S12. Sensitivity analysis according to risk of bias of the association between ApoA1 levels and MetS. Figure S13. Subgroup analysis according to diagnostic criteria of the association between ApoB/ApoA1 ratio levels and MetS. Figure S14. Subgroup analysis according to gender of the association between ApoB/ApoA1 ratio levels and MetS. Figure S15. Subgroup analysis according to continents of the association between ApoB/ApoA1 ratio levels and MetS. Figure S16. Subgroup analysis according to study design of the association between ApoB/ApoA1 ratio levels and MetS. Figure S17. Sensitivity analysis according to risk of bias of the association between [file 12944_2023_1860_MOESM1_ESM.docx]
